# Foundation Model for Biological Temporal Data Dynamics with Experimental Validation

**DOI:** 10.21203/rs.3.rs-9018407/v1

**Published:** 2026-03-12

**Authors:** Xiaoyu Duan, Vipul Periwal

**Affiliations:** 1 Laboratory of Biological Modeling, National Institute of Diabetes and Digestive and Kidney Diseases, National Institutes of Health, Bethesda, MD, USA

**Keywords:** foundation model, latent dynamical systems, variational autoencoder, neural ODE, time series, mechanistic modeling, counterfactual prediction, in silico perturbation, interpretable AI

## Abstract

High-dimensional biological and physiology-adjacent time series are often noisy, partially observed, and heterogeneous across subjects, space, and time, making it difficult to learn continuous-time models that are both stable to integrate and useful for intervention analysis. We propose a reusable latent-dynamics backbone that couples a mask-aware variational autoencoder with a latent neural ordinary differential equation, and returns the learned dynamics to observation space through decoded rollouts or decoder pushforward. In this work, we use the term foundation model in a dynamical sense: a shared transferable backbone for temporal modeling across datasets and downstream tasks. We evaluate the framework on three datasets: 64-channel electroencephalography motor movement and imagery, the AirQualityUCI multivariate environmental time series with meteorological and calendar controls, and a registered *Drosophila* blastoderm gene-expression atlas. Across electroencephalography and air quality, the same backbone improves open-loop forecasting relative to classical baselines and supports controlled counterfactual rollouts. In electroencephalography, it also enables data-efficient subject adaptation. In air quality, it supports interpretable *in silico* intervention screening over exogenous drivers. In the *Drosophila* atlas, the black-box backbone supervises a sparse editable Hill-type mechanistic student model, yielding candidate regulatory structure, equation-level perturbation hypotheses, and an interpretable artificial-intelligence workflow for mechanistic analysis. These results show that a shared latent-dynamics backbone can unify forecasting, adaptation, counterfactual analysis, interpretable AI, and mechanistic distillation across heterogeneous biological time-series settings.

## 1 Introduction

Many biological and health-relevant datasets now arrive as multivariate time series or time-stamped snapshots, including trial-structured electroencephalography (EEG) recordings, environmental exposure measurements, and spatiotemporal single-cell expression atlases. These data are often high dimensional, noisy, partially observed, and heterogeneous across time, subjects, or spatial locations. Across such settings, an important goal is to learn smooth continuous-time dynamics that remain numerically stable under integration, support forecasting beyond the observed samples, and allow systematic *in silico* perturbations that answer practical “what-if” questions. At the same time, it is increasingly valuable to have a single reusable modeling backbone that can be transferred across domains with modest redesign rather than rebuilding a new architecture for each modality.

Existing methods address parts of this problem but often remain tied to particular assumptions, data types, or scientific aims. In single-cell analysis, latent ordinary differential equation (ODE) models and related deep generative approaches learn continuous trajectories in low-dimensional latent spaces^1;2^. Optimal-transport based methods reconstruct population-level dynamics from time-stamped snapshots^3;4^. RNA-velocity based approaches estimate transcriptomic vector fields from spliced and unspliced reads and use them for fate mapping and lineage inference^5–8^. In parallel, ODE-based gene regulatory network inference methods such as SCODE and GRISLI operate directly in gene space with sparsity priors, but they typically rely on relatively dense or well-resolved temporal information^9;10^. Outside genomics, recurrent networks, temporal convolutional networks, and other sequence models are widely used for EEG and environmental sensing, but many emphasize prediction accuracy without explicitly providing stable continuous-time integration, controlled counterfactual rollouts, or interpretable equation-level mechanisms. Meanwhile, so-called foundation-style modeling in biology has advanced rapidly for static representation learning, such as protein language models and large vision encoders, yet a gap remains between static pretrained representations and reusable continuous-time dynamical models that can support forecasting, intervention, and mechanistic reasoning across heterogeneous temporal datasets.

Here we propose a reusable latent-dynamics backbone that couples a mask-aware variational autoencoder (VAE) with a latent neural ordinary differential equation (neural ODE). At a high level, the framework first compresses high-dimensional observations into a compact latent state, then learns continuous-time evolution in that latent space, and finally maps the resulting trajectories back to observation space for prediction and intervention analysis. When exogenous information is available, such as task identity, meteorological covariates, calendar variables, or spatial context, it can be incorporated as a control signal at the dynamical stage. This design yields a single modeling recipe that can be reused across distinct temporal data types while still allowing domain-specific downstream tasks.

In this paper, we use the term *foundation model* in this narrower dynamical sense: not as a web-scale universal model, but as a shared, transferable backbone for temporal dynamics that is trained once in a generic form and then reused across datasets, controls, and scientific questions. Its “foundation” character comes from three properties demonstrated here: first, the same latent representation-and-dynamics architecture is deployed across EEG, air-quality, and developmental gene-expression data; second, the pretrained backbone supports downstream reuse such as subject adaptation and control-conditioned rollouts with limited redesign; and third, the learned latent dynamics can be realized in observation space for forecasting, intervention screening, and mechanistic distillation. Thus, our claim is not that the model is universal in the language-model sense, but that it functions as a domain-agnostic foundation for a family of time-resolved modeling tasks.

The observation-space realization of the learned dynamics is central to this framework. We use two complementary realizations. Decoded rollouts map integrated latent trajectories back to predicted observation trajectories. Decoder pushforward maps the latent vector field through the decoder Jacobian to induce a velocity field in the original variables. Together, these two views allow the latent backbone to act not only as a compact predictor, but also as an engine for *in silico* perturbation screening in measurement space: one can roll trajectories forward under altered controls, initial states, or structural edits and inspect the resulting predicted dynamics. In this sense, the generative component of the framework is not limited to static reconstruction, but extends to producing plausible time-evolving trajectories under hypothetical conditions.

A second goal of this work is to make black-box dynamics more actionable for scientific interpretation. To this end, we attach mechanistic student models that distill structure from the backbone’s denoised observation-space dynamics. In the present study we focus on a Hill-type regulatory ODE in gene space as a student model for the *Drosophila* atlas. We fit this student to the teacher-induced observation-space velocities, obtaining a sparse regulatory system whose couplings appear explicitly in saturating dynamical equations. Because the student is an editable ODE, its edges can be perturbed directly to perform equation-level *in silico* interventions. This teacher–student construction therefore serves as a form of interpretable AI for biology: a black-box dynamical teacher provides stable denoised trajectories and derivative estimates, while a sparse mechanistic student exposes candidate regulatory interactions in a form that can be inspected, pruned, calibrated, and perturbed. We do not claim formal causal identification from observational data alone; rather, we use the student to generate dynamical causal hypotheses and candidate regulatory mechanisms that can be compared against published perturbation experiments and tested prospectively.

We illustrate the framework on three structurally different but conceptually related datasets that each involve multivariate dynamics, heterogeneous missingness, and scientifically meaningful counterfactual questions. First, a 64-channel EEG motor movement and imagery dataset from PhysioNet^11;12^ provides high-frequency neural signals with protocol-defined run types and strong subject variability. Here we train a universal VAE on short overlapping windows pooled across subjects, and then use run-level latent controls at the dynamical stage to enable run-conditioned forecasting, counterfactual rollouts, and efficient subject adaptation from a shared pretrained representation.

Second, the AirQualityUCI dataset^13^ provides a year-long hourly record coupling pollutant channels with meteorological covariates and substantial missingness. We include this dataset as a compact but informative proxy for many biological time-series challenges: coupled multivariate dynamics, structured missingness, and strong exogenous drivers. It provides a lightweight setting for reproducible control-conditioned intervention screens over weather and calendar variables and helps demonstrate that the backbone is not restricted to one biological modality.

Third, a registered fluorescence atlas of *Drosophila melanogaster* blastoderm embryos^14^ provides spatially resolved gene-expression measurements at six time points with characteristic block-missing structure across genes and time. Here the latent backbone addresses temporal sparsity and missingness, while the mechanistic student supplies editable structure in gene space. Prior work on this atlas inferred discrete-time mechanistic networks directly in the 99-dimensional observation space using robust regression^15^. In contrast, we use the latent backbone to generate smooth, variance-reduced derivative estimates and short-horizon predictions that supervise a sparse editable Hill-type ODE, thereby connecting black-box forecasting to interpretable regulatory-network discovery and perturbation screening. To relate these *in silico* interventions to experimental literature, we compare the direction and spatial pattern of selected equation edits with published perturbation outcomes in early blastoderm patterning.

**Fig 1.**
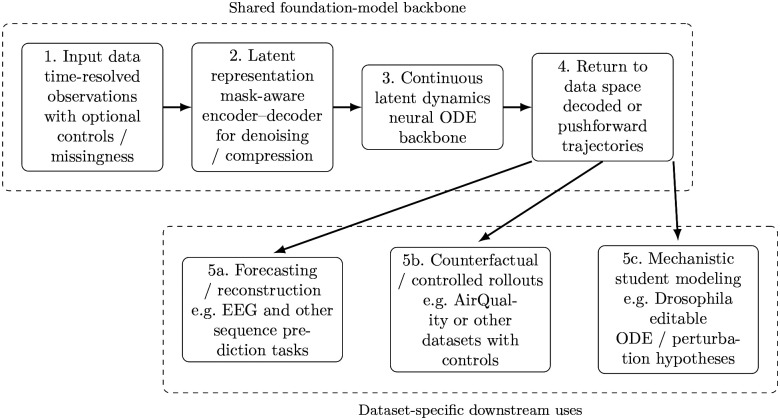
Conceptual workflow of the framework. A shared latent-dynamics backbone first maps high-dimensional time-resolved data into a compact latent representation, learns continuous-time evolution in latent space, and returns trajectories to observation space by decoding or pushforward. The learned backbone is then used in different ways depending on the dataset and scientific goal, including forecasting or reconstruction, controlled or counterfactual rollouts when control variables are available, and mechanistic student modeling for interpretable perturbation analysis. This figure is intended as a high-level roadmap; implementation details are provided in the Methods section.

The conceptual workflow is summarized in Fig. 1. Readers may view this figure as a roadmap for the remainder of the paper: a shared latent-dynamics backbone is first trained on high-dimensional observations, then realized in observation space, and finally used in different downstream modes depending on the dataset, including forecasting, controlled counterfactual rollouts, and mechanistic student modeling. Detailed formulations, training procedures, and implementation choices are provided in the Methods section.

Overall, this work makes three contributions. Conceptually, we formulate a reusable latent dynamical backbone for heterogeneous time-resolved biological and health-related data, and we argue that its cross-domain reuse and downstream adaptability justify viewing it as a foundation model in a dynamical rather than language-model sense. Methodologically, we show how decoded rollouts and decoder pushforward turn latent dynamics into observation-space forecasts, controlled counterfactual analyses, and *in silico* perturbation screens. Scientifically, we demonstrate that mechanistic distillation into a sparse Hill-type ODE yields an interpretable-AI workflow for biology in which editable equations provide candidate regulatory structure and testable dynamical causal hypotheses in the *Drosophila* blastoderm atlas.

## 2 Results

We organize the Results around the practical outputs of the framework shown in Fig. 1, so that readers can follow the scientific use-cases without first needing the full implementation details. We begin with the shared black-box backbone and ask whether it provides accurate and stable open-loop forecasting on EEG and AirQualityUCI relative to classical baselines. We then show that the same pretrained backbone can be reused for downstream tasks, including data-efficient subject adaptation and run-conditioned counterfactual rollouts in EEG, and control-conditioned intervention screens in urban air quality. Finally, we turn to the *Drosophila* blastoderm atlas, where the black-box model serves as a teacher for a sparse Hill-type mechanistic student whose editable equations support interpretable-AI analysis, candidate regulatory-network discovery, and prospective perturbation hypotheses. Readers who wish to inspect exact architectures, loss terms, hyperparameters, and additional ablations can find them in the Methods section and Supplementary Information Text S1.

### 2.1 The shared latent-dynamics backbone improves open-loop forecasting on EEG and AirQualityUCI

We begin by asking a basic question: before using the framework for counterfactual rollouts, intervention screening, or mechanistic distillation, does the shared latent-dynamics backbone already provide strong predictive performance as a forecasting model? To answer this, we evaluated multi-step *open-loop forecasting* on held-out data, meaning that after initialization the model predicts future states recursively without being corrected by future observations. We use the same error metric within each dataset across all compared methods. For motor-imagery electroencephalography (EEG), we report EEG-space mean squared error (MSE), averaged over held-out sequences and all 64 channels. For AirQualityUCI, we report masked MSE, computed only on pollutant entries that are actually measured at the forecasted target time. This benchmarking step serves two purposes: it tests whether the shared backbone is competitive as a general nonlinear forecasting model, and it establishes a predictive foundation for the more intervention-oriented analyses that follow.

Across both datasets, we compare our black-box backbone–a universal variational autoencoder (VAE) encoder–decoder coupled to a latent neural ordinary differential equation (neural ODE)–against standard classical references. These include a first-order vector autoregressive predictor trained directly in observation space (VAR(1)) and a persistence baseline that simply holds the last observed value constant throughout the rollout. For AirQualityUCI, we also include two stronger classical baselines that use external covariates: a control-augmented linear autoregressive model (VARX(1)) using the same meteorological and calendar inputs supplied to our controlled latent neural ODE, and a seasonal autoregressive integrated moving average model with exogenous regressors (SARIMAX), fit separately for each pollutant.

**Fig 2.**
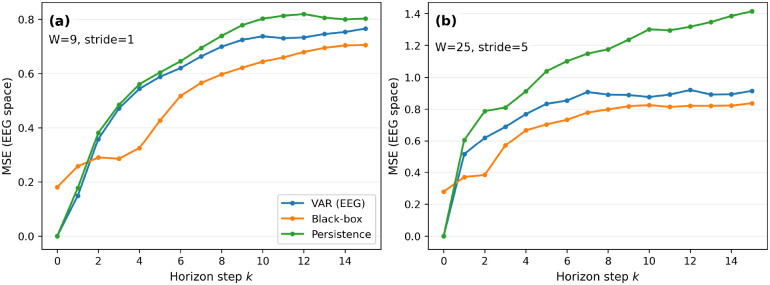
Baseline multi-step forecasting benchmark on motor-imagery EEG. We compare our black-box backbone (universal VAE encoder–decoder coupled to a latent neural ODE) against (i) a first-order vector autoregressive baseline trained in EEG space (VAR(1)) and (ii) a persistence baseline that predicts ${\overset{ˆ}{x}}_{t+k}=x_{t}$ for all k≥0. Methods are evaluated by open-loop rollouts of H=15 horizon steps and EEG-space MSE averaged over held-out validation sequences and all 64 channels. **(a)** W=9,stride=1: the black-box model yields the lowest MSE, while the linear autoregressive baseline is competitive at the shortest horizons under dense sampling. **(b) W=25,stride=5**: the black-box model yields lower MSE and degrades more slowly with horizon, highlighting improved longer-horizon prediction. Horizon step k corresponds to $k\cdot \text{stride}/f_{s}$ seconds with $f_{s}=160\;\text{Hz}$.

On motor-imagery EEG, we first consider a dense short-window regime (W=9,stride=1), which emphasizes very short-term sensor evolution and therefore gives a relatively favorable setting for local linear approximations. Even in this regime, the latent-dynamics backbone achieves the lowest MSE across forecast horizons, although the linear autoregressive baseline remains competitive at the very shortest horizons (Fig. 2a). We then increase the effective forecasting difficulty by using a longer window and coarser stride (W=25,stride=5), which probes longer-range evolution in time. Under this setting, the advantage of the black-box backbone becomes clearer, and its error grows more slowly with horizon than the classical baselines (Fig. 2b). Across both EEG settings, the persistence baseline performs worst and deteriorates fastest with horizon, confirming that the gains are not explained by trivial short-term carryover.

**Fig 3.**
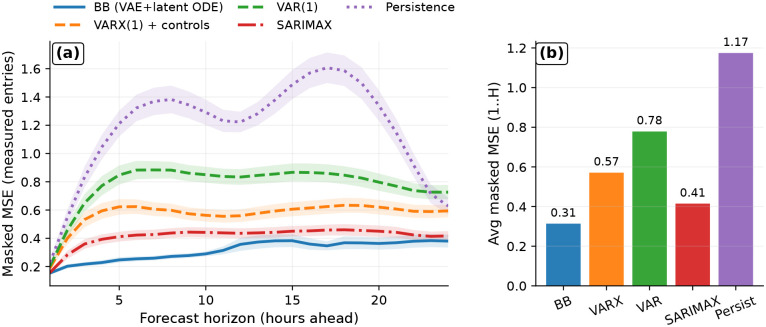
AirQualityUCI baseline forecasting benchmark against classical time-series baselines. We evaluate multi-step forecasting on a held-out set of n=327 valid start times t (window centers) for which the input window is available and the next H=24 hours contain at least one measured pollutant entry under the mask. **(a)** Masked mean-squared error (MSE) on measured pollutant entries versus forecast horizon for our black-box backbone and four baselines: persistence, VAR(1), VARX(1) with matched exogenous controls, and SARIMAX (fit separately per pollutant). Shaded bands indicate uncertainty across forecast starts (mean ± SEM). **(b)** Average masked MSE aggregated over horizons 1: H.

On AirQualityUCI, the black-box backbone achieves the lowest masked MSE across forecast horizons up to H=24 hours (Fig. 3a) and also the lowest error after aggregation over horizons 1: H (Fig. 3b). Adding exogenous covariates to the linear autoregressive model improves performance, indicating that calendar structure and meteorological drivers do carry predictive signal for pollutant evolution. However, the latent-dynamics backbone still outperforms both this control-augmented linear model and the stronger seasonal SARIMAX baseline, supporting the value of a shared nonlinear state representation combined with continuous-time latent dynamics.

When averaged over horizons 1:H, the black-box model attains masked MSE 0.31, compared with 0.41 for SARIMAX, 0.57 for VARX(1), 0.78 for VAR(1), and 1.17 for persistence. Taken together, these results show that the shared backbone is not only transferable across domains but also competitive as a practical forecasting model under realistic missingness and exogenous forcing. This predictive strength motivates its later use for controlled rollouts and intervention screening rather than treating those downstream analyses as unsupported add-ons.

Overall, the forecasting benchmarks establish that the proposed latent-dynamics backbone is already useful as a general predictive model before any mechanistic interpretation is imposed. We therefore next ask whether the same shared representation can be reused efficiently at the subject level in EEG, and whether the learned dynamics can support controlled counterfactual analyses in EEG and air quality.

### 2.2 A universal EEG representation supports accurate reconstruction and efficient subject adaptation

#### A single universal VAE preserves fine-scale EEG structure across subjects.

Having established forecasting performance, we next examine the representation layer itself. Specifically, we ask whether a single universal variational autoencoder (VAE) can reconstruct raw motor-imagery EEG across subjects without subject-specific training. We train the universal VAE on pooled windows from 70 subjects, reserve 10 subjects for validation during training, and hold out 29 subjects for downstream evaluation and adaptation experiments. Using subject S001 under run R01 as an illustrative example, each time point is represented by a 9-sample × 64-channel window. The decoder reconstructs the full window, and we then extract its center sample to obtain a reconstruction aligned to the original EEG time axis. Across all 64 channels, the reconstructed waveforms closely follow the measured activity, including rapid fluctuations and transient oscillatory structure (Fig. S1). A zoomed view of four representative channels likewise shows near pointwise agreement between measured and reconstructed signals (Fig. S2), supporting the use of the learned latent state as a denoised representation for downstream dynamical modeling.

#### The universal representation adapts efficiently to new subjects.

**Fig 4.**
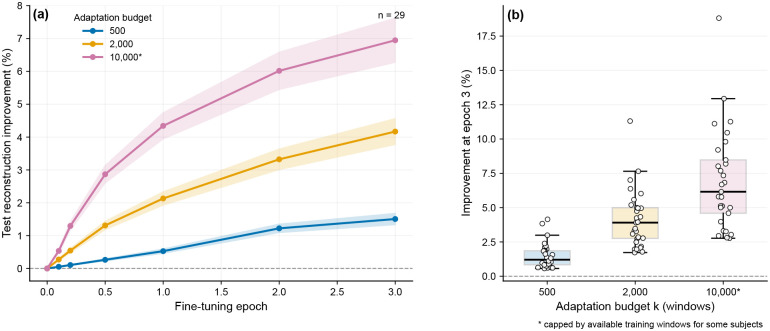
Fine-tuning efficiency for subject adaptation. (a) Mean test-set reconstruction improvement relative to epoch 0 versus fine-tuning epoch for three subject-specific adaptation budgets (k∈{500,2,000,10,000} windows). Shaded bands indicate ± standard error across subjects (n=29). The test set is held fixed for each subject across all k, so differences reflect adaptation data availability rather than evaluation-set variation. (b) Subject-wise distribution of the epoch-3 improvement, using the same metric as in panel (a), for each k, shown as boxplots with overlaid per-subject points. For k=10,000, the adaptation budget is capped by each subject’s available training pool.

We next quantified how quickly this shared representation can adapt to a previously unseen subject. To do so, we performed a controlled fine-tuning experiment on the held-out set of 29 subjects. For each subject, we fixed a subject-specific test set of 2,000 windows and fine-tuned the pretrained VAE using k∈{500,2,000,10,000} subject-specific windows drawn from the remaining pool, while keeping the same test set across all k. Reconstruction quality improves with both fine-tuning epoch and adaptation budget, with larger k yielding faster and larger gains (Fig. 4). Between-subject variability also increases with k, consistent with heterogeneous amounts of subject-specific structure that are not fully captured by the universal model before adaptation. These results support one of the central “foundation” properties claimed in this work: the representation learned from pooled data is reusable, transferable, and data-efficient to specialize.

### 2.3 Run-conditioned EEG dynamics support stable rollouts and counterfactual task controls

#### Short-horizon sensor rollouts remain stable and align with local fluctuations.

**Fig 5.**
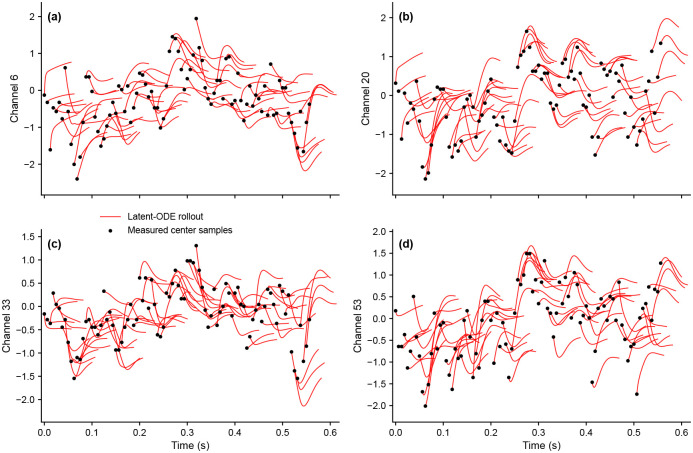
Short-horizon EEG predictions from the universal VAE + latent neural ODE backbone. Panels (a–d) show channels 6, 20, 33, and 53 for a held-out segment from subject S033 under run R01. Black dots indicate measured EEG amplitudes at the centers of consecutive 9-point windows. Red curves show short-horizon decoder-pushforward rollouts obtained by projecting the latent ODE velocity through the decoder Jacobian and integrating forward for 9 steps (≈ 56 ms) from each window center. Across channels, the rollout ensemble remains stable and is broadly consistent with local directional structure in the data.

We next asked whether the learned latent dynamics remain meaningful after being mapped back to the original EEG sensor space. In this experiment, the latent neural ODE is supplied with a 7-dimensional control vector u encoding run identity (R01–R07), while the VAE itself is trained without such controls. Here, “control” means external contextual information that influences the dynamics without being part of the reconstructed EEG signal itself. For a held-out segment from subject S033 under baseline run R01, we initialize the model from consecutive data-derived window centers and integrate forward for 9 steps from each center (≈ 56 ms at 160 Hz). We then obtain sensor-space trajectories by mapping the latent velocity through the decoder Jacobian and integrating the induced velocity in EEG space. Across representative channels, the resulting short-horizon rollout ensemble remains stable and broadly follows the local ups and downs of the measured data (Fig. 5), indicating that the learned latent vector field supports well-behaved short-term prediction in the original sensor coordinates.

#### Counterfactual run controls induce systematic directional changes from matched initial states.

To test whether the model responds meaningfully to contextual changes, we keep the initial EEG state fixed and alter only the run-level control, switching from baseline run R01 to run R03, which corresponds to a different task condition. For each window and channel, we then compute the change in final predicted amplitude after the 9-step rollout, $\Delta x_{\text{final}}=x_{\text{alt}}-x_{\text{base}}$, where $x_{\text{base}}$ and $x_{\text{alt}}$ denote the baseline and counterfactual predictions, respectively. When aggregated across channels and windows, this quantity is shifted below zero (Fig. S3), indicating that the model responds to the changed run context with a systematic directional effect rather than random variation. This provides a concrete example of counterfactual rollout in EEG and illustrates how the shared backbone can be queried under altered task conditions after training.

### 2.4 Control-conditioned pollutant dynamics enable calendar and meteorology perturbation screening

#### Mask-aware representation learning preserves pollutant structure under missingness.

We next turn to AirQualityUCI to test the same framework in a setting with strong exogenous drivers and substantial missingness. After excluding NMHC_GT_ (Non-Methane Hydrocarbons), which is missing for most timestamps in the raw table, we train a masked windowed VAE on 3-hour windows to learn a low-dimensional representation of local air-quality context. The reconstruction loss is computed only on entries that are actually measured, so the model is encouraged to respect the missing-data pattern rather than treating imputed values as ground truth. On a held-out test subset, reconstruction scatter plots show close agreement between measured and reconstructed center values across pollutant variables (Fig. S4), supporting the use of the latent state as a denoised representation despite substantial missingness.

#### Post-VAE gap filling differs from local linear interpolation.

To illustrate what the learned multivariate representation adds in practice, we compare simple per-channel linear interpolation with VAE-based post-reconstruction on local intervals containing large missing gaps. Within the missing intervals, the VAE-based estimates can differ substantially from purely local linear interpolation (Fig. S5), consistent with the model using cross-channel relationships and temporal context from surrounding hours rather than relying only on nearest observed values within a single pollutant channel.

#### Controlled latent dynamics yield stable piecewise trajectories over day-scale horizons.

**Fig 6.**
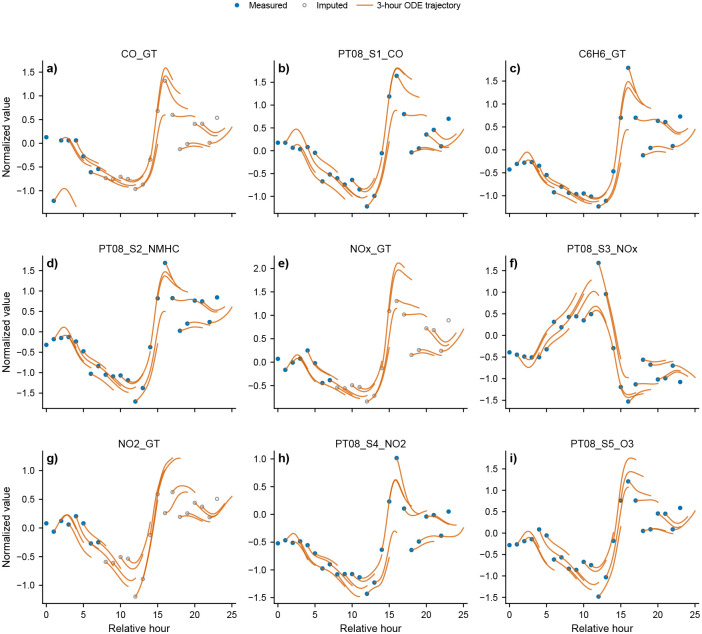
Piecewise 3-hour neural-ODE trajectories via pushforward velocities over a 24-hour window. Dots show normalized pollutant observations (filled: measured, open: imputed). Curves are obtained by decoder pushforward of the latent ODE velocity, $\dot{x}(t)=J_{G}(z(t))\dot{z}(t)$, and integrating the induced velocity field in pollutant space within each 3-hour segment.

We then fit a continuous-time latent neural ODE whose vector field is conditioned on a time-dependent control signal u(t) containing calendar features (hour and day encodings, weekend indicator) together with meteorological covariates. To visualize the resulting dynamics in pollutant space, we map the latent velocity back to observation space using the decoder Jacobian, written as $\dot{x}(t)=J_{G}(z(t))\dot{z}(t)$, where G denotes the decoder. We generate 24-hour trajectories by integrating within successive 3-hour segments and re-initializing each segment from the local observed or reconstructed state, a procedure we refer to as piecewise re-anchoring. The resulting pollutant trajectories remain stable and track observed or imputed values across the day-scale window (Fig. 6), providing an observation-space dynamical system that can be queried under altered controls.

#### Temperature-swap intervention produces interpretable shifts in predicted local tendencies.

To probe sensitivity to meteorological controls, we perform an *in silico* intervention in which only the temperature component of u(t) is altered: for January hours, we replace it with the July temperature at the same day and hour, while keeping all other control variables fixed. We then evaluate the corresponding change in the instantaneous pushforward velocity in pollutant space, summarized as $\Delta \dot{x}$ in Supplementary Fig. S15. This provides a simple intervention screen along an interpretable environmental axis and shows that the predicted local pollutant tendencies shift in a channel-specific manner under a warmer temperature scenario, without retraining the model.

### 2.5 Latent-dynamics teacher enables sparse Hill-type mechanistic modeling in the *Drosophila* atlas

#### Teacher–student transfer from black-box dynamics to a sparse Hill network.

**Fig 7.**
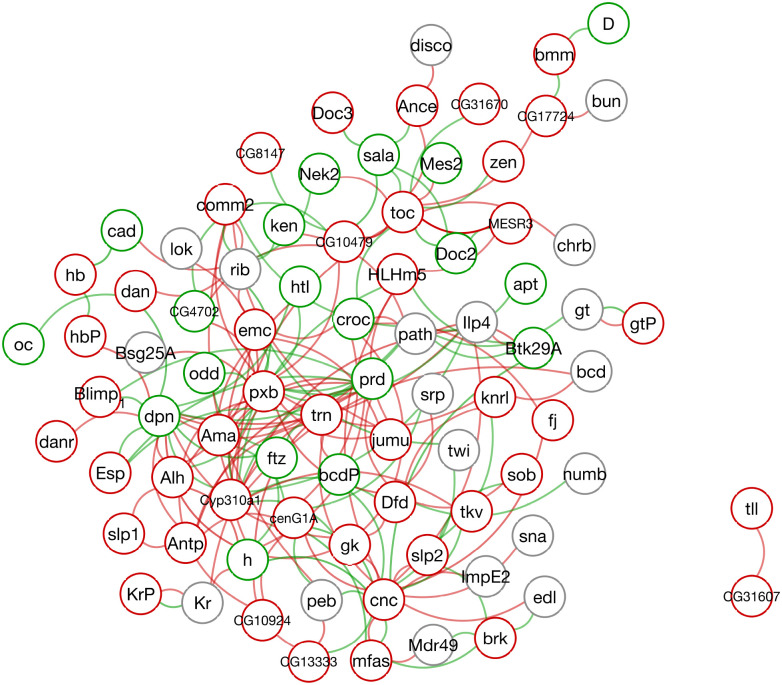
Sparse Hill network as an interpretable regulatory graph. Nodes denote genes, with outline color indicating the sign of self-regulation (green: positive, red: negative, gray: none). Directed edges represent fitted couplings $V_{ij}$, drawn from regulator j to target i. Green edges indicate activation ($V_{ij}>0$), red edges indicate repression ($V_{ij}<0$), and transparency scales with coupling strength. The sparse structure arises from $\ell_{1}$-regularized fitting over Hill-function terms and provides an interpretable summary of candidate directed influences, including the inferred repressive effect of gt on eve.

We next turn to the *Drosophila* blastoderm atlas, where only six developmental time points are observed and many genes have structured block-missing measurements. This is the setting in which we move beyond forecasting alone and ask whether the black-box backbone can support interpretable mechanistic modeling. Here the backbone provides two useful outputs: refined gene-expression tables through mask-aware reconstruction, and smoothed local gene-space dynamics obtained from observation-space realizations of the learned latent system. We then fit a Hill-type regulatory ordinary differential equation (ODE) as a mechanistic student under sparsity constraints so that it approximates these teacher-derived local dynamics. The resulting interaction matrix V can be visualized as a directed regulatory graph (Fig. 7), providing an interpretable summary of candidate directed influences that are consistent with the learned dynamics in the observed developmental regime.

#### Observation-space realizations of the teacher provide two complementary supervision signals.

We use two realization modes of the black-box dynamics to obtain gene-space information suitable for mechanistic fitting. In a *pushforward* realization, the latent velocity is mapped through the decoder Jacobian to yield an observation-space velocity directly at the encoded state. In a *decoded-rollout* realization, the latent ODE is integrated across each interval and the resulting latent path is decoded into a dense observation-space trajectory, from which derivatives are computed on a fine time grid. The two signals are therefore related but not identical. In practice, pushforward starts exactly from the observed or reconstructed state at the segment boundary and thus avoids boundary reconstruction mismatch, whereas decoded rollouts provide a smooth within-interval trajectory. We summarize protocol details and additional qualitative comparisons in the Supplementary Information and use these teacher signals below to assess the impact of teacher guidance on mechanistic fitting.

#### Interpretable regulatory structure highlights candidate influences including gt→eve.

The sparse structure of V yields a compact network-level view of activation and repression relationships. Because each edge corresponds to an explicit term in the Hill equations, the fitted model supports targeted equation-level edits within the chosen mechanistic hypothesis class. This makes the student model useful for interpretable-AI analysis: it does not merely score candidate interactions, but embeds them in editable dynamical equations. We focus below on the inferred repressive influence of *giant* (gt) on *even-skipped* (eve) as a concrete case study for mechanistic intervention and dynamical causal hypothesis generation.

### 2.6 Teacher guidance improves or matches mechanistic fitting while preserving interpretability

We next asked whether the learned black-box teacher actually improves downstream mechanistic fitting, rather than merely providing a more elaborate preprocessing stage. Because the teacher is optimized for reconstruction and dynamical consistency in its own latent space, there is no automatic guarantee that it will provide a better supervision signal for a sparse Hill-network ODE. We therefore compared two matched regimes: a *teacher-guided* regime, in which a VAE plus latent neural ODE provides gene-space supervision signals, and a *data-only* regime, in which the Hill model is fit without such guidance. In both cases, we evaluate the same one-step forecast task (t→t+1) on a fixed test set.

We restrict to G=27 genes with complete measurements across all six time points to avoid imputed ground truth and to keep supervision windows identical across regimes. Both pipelines enforce the same sparsity and interpretability constraints: an $\ell_{1}$ penalty on the interaction matrix V, hard pruning of small entries, and a fixed interaction structure thereafter. After pruning, we freeze the interaction support ℰ (the directed gene–gene graph) and the coefficients $V_{ij}$; a subsequent calibration stage then tunes only $\alpha ,\gamma ,b_{0}$, and the Hill shape parameters $\left(K_{ij},n_{ij}\right)$ for (i,j)∈ℰ without altering ℰ or V. Predictions are obtained by integrating the ODE over a single interval.

Under these matched conditions, results for G=27 fully observed genes (W=1) show that teacher-guided fits achieve equal or lower error after calibration, with the clearest advantage under stronger sparsity (Table 1). Thus, the black-box teacher is not only compatible with mechanistic interpretability but can also improve, or at minimum preserve, mechanistic predictive quality while maintaining a compact editable graph.

Following the definitions in the Methods, Fig. S8 contrasts two black-box realizations over each interval $\left[t_{k},t_{k+1}\right]$: **(a)** the *pushforward gene-space ODE* integrated from the observed $g\left(t_{k}\right)$, and **(b)** the *decoded latent trajectory* obtained by decoding a path in latent space. For Fig. S8 only, we ablate the latent dynamics in **(b)** and use linear interpolation between $\mu \left(t_{k}\right)$ and $\mu \left(t_{k+1}\right)$ before decoding. This shows how the VAE itself performs where we set latent dynamics to be trivial. Pushforward starts exactly at the observed state and therefore avoids reconstruction error at the segment boundary, whereas decoded latent paths provide a smooth within-interval realization.

In Fig. S9, we compare mean absolute prediction errors across 27 fully measured genes between our black-box model, the Hill model, and the erf-weighted LAD models of^15^. While the LAD quadratic model achieves the lowest raw error, our black-box approach remains competitive, and the Hill fits guided by the black-box dynamics offer a complementary advantage: they embed regulatory interactions directly in mechanistic Hill-type nonlinearities, rather than being limited to linear or quadratic couplings. Although the mean errors of our models are modestly higher, they produce interpretable functional forms that can be directly manipulated and biologically motivated, thereby providing a framework well-suited for hypothesis generation and experimental design.

Finally, we return to the full 99-dimensional gene space, where missingness makes direct mechanistic fitting substantially more difficult. In this setting, we first train the black-box teacher and then fit the Hill model using VAE-based imputation to supply missing entries needed for derivative estimation and one-step targets. Although the *teacher-guided* Hill fit does not dominate the *data-only* version in every setting, the deep-learning stage remains essential because it makes the full-network fit feasible under partial observation. Thus, even when teacher guidance does not provide the strongest direct supervision signal, the black-box stage still contributes crucially by restoring the information needed to formulate the mechanistic fitting problem in the full 99-gene space.

To visualize how teacher guidance shapes downstream mechanistic fits, Fig. S10 overlays continuous trajectories produced by the black-box model (VAE with latent neural ODE; blue) and the corresponding Hill-field rollouts (red) directly in the 99-dimensional gene space between observed time points. Solid blue dots indicate measured expressions and hollow circles indicate VAE-imputed entries at the same states. Subplots titled in green are the 27 fully observed genes. This comparison emphasizes that the teacher and student can remain qualitatively aligned even in the full partially observed setting.

### 2.7 Equation-level interventions isolate regulatory routes in a gt→eve case study

#### Absolute patterns under baseline integrations and mechanistic edits.

**Fig 8.**
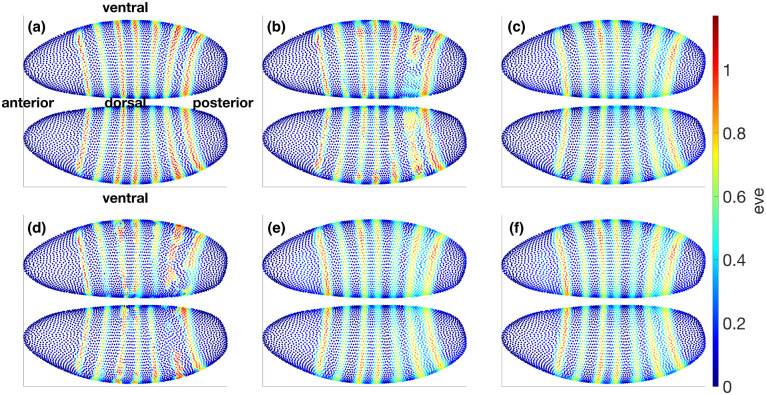
Absolute eve patterns under baseline and mechanistic operations. **(a)** Ground truth at t=4. **(b)** Black-box baseline integration. **(c)** Hill baseline integration. **(d)** Black-box with gt knockout. **(e)** Hill with gt knockout. **(f)** Hill with the single edge $V_{gt\to eve}$ removed while all other couplings are intact. A common color scale is used across all panels.

We next illustrate how the mechanistic student can be used for explicit equation-level intervention analysis. We examine eve as a sensitive downstream readout and gt as an upstream regulator because their interaction is prominent in the learned network and is also broadly consistent with prior developmental biology. Starting from t=2, we integrate to the target time t=4 under several conditions and visualize the resulting eve pattern on a shared color scale (Fig. 8). Panel **a** shows the atlas ground truth at t=4. Panels **b** and **c** show the corresponding baseline integrations from the black-box teacher and the Hill field, respectively. Panels **d** and **e** apply a gt knockout during integration in the black-box teacher and the Hill model. Panel **f** shows a more specific mechanistic edit in the Hill model, in which only the single edge $V_{gt\to eve}$ is removed while all other couplings are kept fixed. Together, these matched conditions let us compare broad black-box perturbation with targeted mechanistic intervention.

### Difference maps reveal coherent repression relief in the mechanistic model.

**Fig 9.**
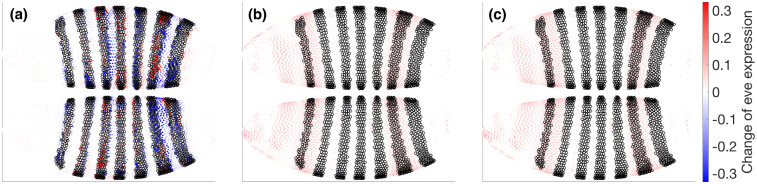
Operation–baseline differences highlight relief of gt-mediated repression. Signed differences (operation minus baseline) for: **(a)** black-box gt knockout minus black-box baseline, **(b)** Hill gt knockout minus Hill baseline, **(c)** Hill removal of $V_{gt\to eve}$ minus Hill baseline. All subplots share the same color scale. Broad positive changes away from stripe peaks indicate increased eve under reduced gt input, while muted changes near stripe maxima and selected gaps suggest compensating inputs and local balance.

To isolate intervention effects, we compute operation–baseline differences at t=4 using a shared color scale (Fig. 9). The Hill knockout and the Hill single-edge edit produce closely matching positive shifts in eve away from stripe peaks, consistent with relief of gt-mediated repression. Their close resemblance suggests that, over the t=2→4 interval and within the present mechanistic hypothesis class, the dominant route by which gt influences eve is the direct gt→eve coupling rather than a more distributed indirect pathway. By contrast, the black-box knockout can show mixed-sign responses because setting gt to zero perturbs the encoded system state globally, moving the dynamics away from the typical learned manifold rather than removing a single signed mechanistic term. These results illustrate the complementary roles of the two model levels: the black-box teacher supplies stable denoised dynamics, whereas the Hill student exposes an editable equation-level interface for interpretable intervention and dynamical hypothesis generation.

### 2.8 Prospective perturbation hypotheses and generalization analyses from the sparse Hill network

#### Dose-response predictions from single-edge scaling.

Because the Hill network is sparse and explicitly parameterized, it also supports graded equation-level perturbations rather than only all-or-none edits. For example, scaling $V_{gt\to eve}$ by factors λ∈{0.25,0.5,0.75} generates a dose-response family of predicted eve patterns at t=4, interpolating between the fitted baseline and the full edge-removal condition. The resulting predictions suggest that partial relief of gt-mediated repression first elevates eve in inter-stripe troughs before broader stripe reshaping appears under stronger reductions.

#### Combinatorial mechanistic edits yield multi-perturbation hypotheses.

Beyond single edges, the mechanistic form enables combinatorial perturbations by jointly editing a small set of couplings into a target gene, producing explicit spatial hypotheses for how particular stripe features weaken, shift, or broaden under compound changes. Such multi-edge edits correspond to hypothetical compound perturbations and can be used to prioritize candidate regulatory routes for follow-up experiments.

#### Balanced spatial coverage improves generalization of region-restricted mechanistic fits.

A practical question for reuse is whether mechanistic fits trained on restricted spatial subsets generalize across the embryo. To assess the spatial universality of the mechanistic model inferred from our learned latent dynamics, we examined how restricting supervision to contiguous regions of the embryo affects prediction accuracy across the full embryo. This parallels the regional training experiments in^15^, but here we apply it to Hill fields fitted to gene-space velocities produced by the black-box teacher. For consistency, we did not retrain the VAE with a neural ODE but instead used the trivial latent linear dynamics together with the VAE pushforward to obtain gene-space derivatives.

We partitioned the embryo along either the anterior–posterior axis into three equal-sized regions (anterior, middle, posterior; AMP) or along the dorsal–ventral axis into dorsal, medial, and ventral (DMV) subsets (Fig. S11). Each region covers roughly one third of the positional bins after test-set removal, i.e. about 33% of the training pool (the 90% of bins not in the fixed 10% test set).

For each region, we trained a Hill field using only that region’s bins across all time points, following the same $\ell_{1}$-regularized training and relative-threshold pruning procedure used elsewhere. To ensure that differences across regions reflect supervision alone rather than variability in upstream representation learning, we did not retrain a separate latent neural ODE for each case. Instead, we derived gene-space derivatives directly from a fixed VAE pushforward with trivial latent dynamics, so that all regional Hill models are trained from a common, consistent source of velocities. For comparability, we also omit the final calibration step here, reporting raw regional fits without further adjustment.

As a baseline, we also trained “random-30%” models by uniformly sampling 30% of bins from the training pool—the same fraction as in each regional case (AMP or DMV). This ensures that both region-specific and random baselines use equal training size. To reduce variance from sampling, we repeated the random-30% selection with 10 different seeds and averaged the resulting errors. Prediction quality throughout this analysis is reported in terms of the relative error, defined as $|g-\overset{ˆ}{g}|/(|g+\overset{ˆ}{g}|+\varepsilon )$, where g denotes the ground-truth gene expression and $\overset{ˆ}{g}$ the corresponding prediction obtained by integrating the Hill model. This normalization prevents highly expressed genes from dominating the error metric and ensures comparability across genes of different magnitudes.

Fig. S12 and S13 summarize per-gene prediction errors under the two regularization regimes. At $\lambda =10^{-4}$ (stronger regularization), all regional models achieve broadly similar accuracy, but the random-30% baseline provides a small yet consistent advantage. At $\lambda =10^{-5}$ (weaker regularization), this advantage becomes more pronounced: random-30% models outperform any single-region training, showing that broad coverage is particularly valuable when the network is less constrained and retains more couplings. These results indicate that balanced sampling across the embryo improves generalization, with the benefit becoming clearer as regularization is relaxed.

As shown in Fig. S14, the spatial overlays make clear that region-restricted supervision produces non-uniform error distributions across the embryo. For AMP training, anterior- and posterior-trained models tend to overfit locally, leaving elevated error bands in the opposite poles, while the middle-trained model shows weaker generalization in both anterior and posterior extremes (panels a–c, left). Similarly, under DMV supervision, dorsal- and ventral-trained models concentrate error in the opposite domain, and medial supervision underfits in both dorsal and ventral territories (panels a–c, right). By contrast, the random-30% baseline yields a more spatially uniform and consistently lower error profile across the entire embryo (panel d), suppressing the high-error pockets seen in the regional cases. This advantage is particularly evident in posterior and ventral domains, which are most prone to overfitting under single-region training. Crucially, the improvement is not restricted to a handful of genes or cells but extends across the full 27-gene set and all spatial positions, underscoring the benefit of balanced sampling for generalization.

#### General perturbation-map recipe for future datasets.

More broadly, for any sufficiently strong regulator–target coupling, one can set $V_{ij}=0$, weaken it, or strengthen it, and then integrate forward from a chosen initial condition to generate operation–baseline difference maps analogous to Fig. 9. This provides a systematic way to turn a black-box dynamical teacher into a compact catalogue of spatially resolved perturbation predictions through an interpretable mechanistic student. When matched perturbation data are available, the same equation-level edits can be compared directly with observed outcomes, turning these mechanistic manipulations into testable dynamical causal hypotheses and a practical tool for perturbation-study design.

## 3 Discussion

This study supports the view that a single latent-dynamics backbone can serve as a reusable dynamical engine across heterogeneous biological and physiology-adjacent time series. By combining a mask-aware variational autoencoder with a latent neural ordinary differential equation and then mapping the learned dynamics back to observation space, the framework provides a common route to denoising, forecasting, control-conditioned rollout, and mechanistic follow-up across datasets that differ sharply in modality, sampling structure, and missingness pattern. In the present work, the same overall recipe was instantiated on motor-imagery EEG, urban air quality, and the Drosophila blastoderm atlas, yet remained useful in each setting without changing the central modeling logic.

Two points are particularly important for the foundation-model framing. First, the backbone is not only competitive as a forecasting model, but also reusable as a representation-and-dynamics substrate. On EEG and AirQualityUCI, the black-box backbone outperformed or matched strong classical baselines in held-out multi-step forecasting, establishing that the latent continuous-time formulation is practically effective rather than only conceptually attractive. On EEG, the universal representation further supported data-efficient subject adaptation, indicating that the shared encoder–decoder is not merely pooled training for convenience but a transferable representation that can be tuned to unseen subjects with modest additional data. Together, these results strengthen the claim that the model functions as a reusable temporal backbone rather than as a narrowly optimized predictor for a single dataset.

Second, controllable dynamics are a first-class output of the framework. In EEG, run-conditioned latent dynamics produced stable short-horizon sensor-space rollout ensembles from held-out window centers and supported counterfactual run-control changes from matched initial states. In AirQualityUCI, the same general architecture supported control-conditioned day-scale trajectories and intervention screens over interpretable exogenous covariates such as calendar structure and temperature. These examples matter because they move the framework beyond static representation learning or one-shot forecasting. The model can instead be queried under modified conditions while keeping the initial state fixed, which is often the more scientifically relevant operation when the goal is to ask structured “what-if” questions.

The Drosophila atlas provides the strongest illustration of why this backbone is useful scientifically rather than only predictively. In that setting, the black-box model acts as a teacher that supplies denoised expression tables, observation-space realizations of local dynamics, and short-horizon predictive structure in a regime with sparse temporal sampling and block-missing measurements. Distilling this information into a sparse Hill-type student yields a compact, editable mechanistic model whose couplings are explicit and therefore directly perturbable. This teacher–student construction is central to the paper’s broader message: flexible latent dynamics can absorb noise, incompleteness, and nonlinear structure, while the mechanistic student can convert that smoothed dynamical signal into interpretable regulatory hypotheses. The resulting division of labor is pragmatically useful. The teacher contributes robustness and control-aware forecasting, whereas the student provides a native-variable interface for equation-level edits, causal interpretation, and prospective perturbation design.

This perspective also clarifies what we mean by a foundation-style model in this context. The contribution is not a giant pretrained model in the language-model sense, but a reusable architecture-and-training recipe for temporal dynamics that can be ported across domains while retaining several desirable outputs: continuous-time forecasting, control-conditioned rollout, observation-space vector fields, and downstream mechanistic supervision. The EEG adaptation experiments are particularly relevant here because they show that reuse is not only cross-domain in spirit, but can also operate within domain through efficient subject-specific refinement. At the same time, the AirQuality and Drosophila results show that the same backbone can support intervention-oriented analyses in settings with structured missingness and strong exogenous effects.

More broadly, the work suggests a route for connecting black-box dynamics to interpretable scientific modeling without forcing the entire problem into one hypothesis class from the beginning. Many biological systems are too noisy, incomplete, or weakly sampled to fit mechanistic equations directly with confidence. In such settings, a latent continuous-time teacher may be useful precisely because it regularizes the local dynamical signal before mechanistic fitting is attempted. The mechanistic model then becomes a second-stage hypothesis class rather than the only modeling layer. This is especially valuable when the scientific aim is not only prediction error minimization but also the production of compact dynamical hypotheses that can be edited, compared to prior biology, and used to prioritize perturbation experiments.

Several caveats remain, but they are best viewed as boundary conditions on interpretation rather than as a separate limitations section. The backbone is most reliable over horizons that remain reasonably close to the regime represented during training; as the integration horizon lengthens, accumulated latent and decoder errors can degrade realism. Observation-space pushforward dynamics also depend on local decoder geometry, so strong curvature or poorly calibrated latent uncertainty can magnify latent errors after projection back to the original variables. In the control-conditioned analyses, counterfactual outputs should be interpreted as model-based responses of a learned conditional dynamical system, not as guaranteed causal effects in the real world, especially when controls push the state toward regions of weak training support. Likewise, the Hill student is limited by its hypothesis class: it captures sparse saturating regulation and supports editable interventions, but it may miss delays, transport effects, hidden variables, or switching behaviors that fall outside a simple Hill-type ODE representation. These considerations do not negate the framework’s utility, but they do define the regimes in which its perturbation outputs should be treated as prioritized hypotheses rather than definitive quantitative forecasts.

Several future directions follow naturally. One is uncertainty propagation from the encoder and latent dynamics into rollout envelopes, perturbation screens, and mechanistic fits. Another is extension of the student-model layer beyond Hill equations to alternative interpretable classes better matched to particular systems, including low-rank interaction models, piecewise-linear regulatory dynamics, or reaction-network reductions. A third is stronger prospective validation, where counterfactual controls or equation-level edits are ranked before experiment and then evaluated against newly collected perturbation data. In that sense, the present paper should be viewed less as a final mechanistic answer for any one dataset than as a general workflow for turning multivariate temporal data into stable continuous-time predictions, structured in silico interventions, and explicit mechanistic hypotheses.

Overall, these findings support a unified claim: a reusable latent-dynamics backbone can bridge three levels of analysis that are often separated in practice—representation learning, controlled continuous-time forecasting, and interpretable mechanistic hypothesis generation. By showing this across EEG, environmental time series, and developmental gene expression, we argue that the framework is useful not only as a predictive model but also as an experimental design tool for temporal biology and related high-dimensional dynamical datasets.

## 4 Methods

### 4.1 Overview of the modeling framework

Our goal was to build a reusable dynamical modeling framework for high-dimensional, partially observed, time-resolved data. The framework has three conceptual stages. First, a variational autoencoder (VAE) learns a low-dimensional latent representation of high-dimensional observations while respecting missing-data masks. Second, a latent neural ordinary differential equation (neural ODE) learns continuous-time dynamics for the latent states, optionally conditioned on external control variables. Third, the learned latent dynamics are returned to observation space either by decoding integrated latent trajectories or by pushing the latent vector field through the decoder Jacobian. For the *Drosophila* atlas, these observation-space dynamics are then used to supervise a sparse mechanistic student model in gene space.

Throughout, we denote an observation-space state by $x\in R^{d_{x}}$, where $d_{x}$ is the number of measured variables in the dataset. Examples include $d_{x}=64$ for electroencephalography (EEG) channels, pollutant channels for AirQualityUCI, and gene-expression variables for the *Drosophila* atlas. The corresponding latent state is denoted by $z\in R^{d_{z}}$, where $d_{z}\ll d_{x}$. The encoder is denoted by E, the decoder by G, and the latent vector field by $f_{\theta }$, where θ denotes the neural ODE parameters. When external or contextual inputs are used, they are denoted by u(t), where t is time.

At a high level, the framework proceeds as follows.

1. Learn a latent representation z from observations x using a mask-aware VAE.
2. Use the latent states to train a continuous-time latent dynamical system
$$\frac{dz}{dt}=f_{\theta }\left(z\left(t\right),u\left(t\right),t\right),$$
  where the control u(t) is included only when appropriate for the dataset.
3. Map latent trajectories back to observation space either by decoding
$$\overset{ˆ}{x}\left(t\right)=G\left(z\left(t\right)\right),$$
  or by pushing the latent velocity through the decoder Jacobian
$$\frac{dx}{dt}=J_{G}\left(z\left(t\right)\right)\frac{dz}{dt},$$
  where $J_{G}(z)$ is the Jacobian matrix of G with respect to z.
4. For the *Drosophila* dataset, use the resulting observation-space trajectories or velocities as teacher signals for a sparse mechanistic student model.

This section describes those components in detail, followed by dataset-specific instantiations.

### 4.2 Datasets and preprocessing overview

We used three datasets chosen to span distinct modalities while sharing the main challenges targeted by the framework: high-dimensional observations, temporal structure, missingness or heterogeneity, and scientifically meaningful counterfactual questions.

#### Motor-imagery EEG.

We used the EEG Motor Movement/Imagery dataset from PhysioNet^11;12^. This dataset contains 64-channel scalp electroencephalography (EEG) recorded at 160 Hz with the BCI2000 system while subjects perform baseline, executed-movement, and imagined-movement tasks. We treated seven protocol-defined run types (R01–R07) as distinct experimental conditions.

Each run was represented as a multivariate time series of 64 sensor amplitudes. Unless otherwise noted, each time point was encoded by a 9-sample × 64-channel local window, corresponding to 56 ms of data, flattened into a 576-dimensional vector. Windows were constructed with stride 1 for the default reconstruction and short-horizon dynamical analyses. Channel-wise normalization was applied before model fitting. The universal VAE was trained on pooled windows from multiple subjects and all run types without subject-specific labels or subject-specific fine-tuning. In the experiments reported here, 70 subjects were used for training, 10 for validation during training, and 29 were held out for downstream evaluation and adaptation experiments. For the VAE stage, the inputs are short windows of raw EEG, whereas for the dynamical stage the latent neural ordinary differential equation (neural ODE) is optionally conditioned on run identity.

#### AirQualityUCI.

We used the AirQualityUCI dataset from the UCI Machine Learning Repository^13^, which contains hourly chemical-sensor responses and meteorological measurements collected in an Italian city from March 2004 to February 2005. The original table includes variables related to carbon monoxide (CO), benzene, nitrogen oxides (NO_x_), ozone-related sensor channels, and standard meteorological covariates, together with coded missing values. Because one variable, NMHC_GT_, is extremely sparse in the raw dataset, we excluded it from all analyses. After preprocessing, we retained 12 continuous variables: nine pollutant-related channels and three meteorological covariates.

This dataset provides a year-long multivariate time series with substantial but structured missingness. Before model fitting, all retained variables were normalized, and missing entries were pre-filled by simple linear interpolation in time to provide valid numeric inputs to the encoder. These pre-filled values were used only as placeholders and were not treated as observed targets in the loss, which remained mask-aware. Inputs to the VAE were 3-hour local windows extracted from the multivariate time series. The resulting latent representations were then used to train a latent neural ODE. For the dynamical model, the external controls u(t) consisted of calendar-derived quantities together with meteorological covariates, enabling controlled forecasting and intervention analyses.

We evaluated the model using two held-out test protocols. First, to assess robustness across seasonal regimes and sustained missingness patterns, we used a seasonal protocol based on contiguous 24-hour blocks sampled across the year. Second, to assess average-case forecasting performance under the same preprocessing pipeline, we used a random split of time blocks into training, validation, and test subsets.

#### *Drosophila* blastoderm gene-expression atlas.

We used the registered single-cell fluorescence atlas of *Drosophila melanogaster* blastoderm^14^, which consists of measurements from 1,822 embryos registered onto a common spatiotemporal grid of 6,078 positional bins. The atlas contains expression measurements for 95 messenger RNAs (mRNAs) and 4 proteins, which we refer to collectively as 99 genes throughout. The measurements span the approximately 50-minute period preceding gastrulation and are uniformly discretized into six developmental time points t=0,…,5. Among these genes, 27 are measured at all six time points, whereas the remaining 72 exhibit block-missingness and are primarily observed at later time points.

For each sample, the gene-expression vector was optionally concatenated with the corresponding spatial coordinates, which were treated as static context rather than reconstruction targets. Unless otherwise noted, we followed the 90/10 positional-bin split of^15^, with 608 bins held out as a fixed test set. Missing gene entries were initially filled by random sampling within the observed range of each gene and were subsequently refined by a VAE-based fixed-point procedure. We adopted random-fill initialization because it yielded the best held-out reconstruction among the pre-filling schemes tested (Supplementary Fig. S2). This dataset is the main setting in which we combine the black-box latent-dynamics backbone with an interpretable mechanistic student model in gene space.

For each dataset, additional architecture choices, latent dimensions, hidden sizes, optimization settings, and regularization parameters are summarized in Tables S1–S3 where applicable, but the dataset-specific preprocessing and split details above are part of the core experimental design and are therefore stated explicitly here.

### 4.3 Mask-aware variational autoencoder

#### 4.3.1 Basic formulation

The VAE maps a high-dimensional observation x to a latent random variable z. Given an input x, the encoder E outputs the parameters of an approximate posterior distribution

$$q_{\phi }\left(z|x\right)=𝒩\left(\mu_{\phi }\left(x\right),\text{diag}\left(\sigma_{\phi }^{2}\left(x\right)\right)\right),$$

where ϕ denotes encoder parameters, $\mu_{\phi }(x)\in R^{d_{z}}$ is the latent mean, and $\sigma_{\phi }(x)\in R^{d_{z}}$ is the latent standard deviation. A latent sample is then drawn using the reparameterization trick

$$z=\mu_{\phi }\left(x\right)+\sigma_{\phi }\left(x\right)⊙\varepsilon ,\;\varepsilon \sim 𝒩\left(0,I\right),$$

where ⊙ denotes elementwise multiplication. The decoder $G_{\psi }$, abbreviated as G when the decoder parameters ψ need not be emphasized, maps z back to observation space:

$$\overset{ˆ}{x}=G\left(z\right).$$

The VAE objective is the usual reconstruction-plus-regularization objective

$${ℒ}_{\text{VAE}}={ℒ}_{\text{rec}}+\beta {ℒ}_{\text{KL}},$$

where ${ℒ}_{\text{rec}}$ is the reconstruction loss, ${ℒ}_{\text{KL}}$ is the Kullback–Leibler divergence from the approximate posterior to the standard normal prior, and β is the relative weight on the KL term. The KL term is

$${ℒ}_{\text{KL}}=\frac{1}{2}\sum_{j=1}^{d_{z}}\left(\mu_{j}^{2}+\sigma_{j}^{2}-\text{log}\;\sigma_{j}^{2}-1\right).$$

#### 4.3.2 Mask-aware reconstruction loss

Several datasets used here contain missing entries. To avoid training the decoder against unobserved targets, we used mask-aware reconstruction losses.

Let $m\in \{0,1{\}}^{d_{x}}$ denote the binary mask for a single target vector, where $m_{i}=1$ indicates that variable i is observed and $m_{i}=0$ indicates that it is missing. Then the masked mean-squared reconstruction loss is

$${ℒ}_{\text{rec}}=\frac{\sum_{i=1}^{d_{x}}m_{i}{\left({\overset{ˆ}{x}}_{i}-x_{i}\right)}^{2}}{\sum_{i=1}^{d_{x}}m_{i}+\varepsilon },$$

where ε>0 is a small constant preventing division by zero. This ensures that only observed entries contribute to the loss. When the input consists of a window rather than a single vector, the same idea is applied over all entries of the target tensor, again restricting the loss to measured entries only.

#### 4.3.3 Windowed inputs

For EEG and AirQualityUCI, the VAE is trained on short local windows rather than isolated time points. If the window length is W, then a local observation centered at time t is represented as

$$X_{t}=\left[x_{t-(W-1)/2},\ldots ,x_{t},\ldots ,x_{t+(W-1)/2}\right].$$

For even if the implementation stores windows in flattened form, conceptually $X_{t}$ is a local tensor containing multiple adjacent observations. The VAE encodes the window and decodes either the full window or its center depending on the dataset-specific setup. This windowed formulation allows the latent representation to capture local temporal context while remaining time agnostic at the encoder stage.

### 4.4 Pre-imputation and post-VAE imputation

Because the datasets differ in their raw missing-data structure, we distinguish two roles of imputation.

#### Pre-imputation.

A lightweight pre-imputation step may be used only to make the input numerically well formed for neural network processing. For example, simple local interpolation or fill values can be used to populate placeholders in raw arrays before masking. These values are not treated as ground truth, because the reconstruction loss is still restricted to observed entries through the mask. Thus, pre-imputation serves as numerical scaffolding rather than supervision.

#### Post-VAE imputation.

After training, the decoder provides context-aware reconstructed values for all entries, including those that are missing in the raw data. These reconstructed entries are used as post-VAE imputations. Unlike local linear interpolation, post-VAE imputations are informed by multivariate correlations and the learned low-dimensional latent representation. In both the AirQualityUCI and *Drosophila* analyses, these VAE-based reconstructions were used as refined values in downstream stages, including dynamical modeling and comparison with simpler imputation strategies.

#### Fixed-point refinement of post-VAE imputations.

For datasets with substantial or structured missingness, we further applied an iterative post-VAE refinement procedure to obtain self-consistent filled values. This was used in both the AirQualityUCI and *Drosophila* analyses. Starting from an initial table in which observed entries are kept fixed and missing entries are filled by decoder reconstructions, we repeatedly re-encode and re-decode the table while clamping observed entries to their measured values. This procedure seeks a self-consistent latent representation whose decoded output agrees with observations where available and yields stable filled values where data are missing. We refer to this iterative procedure as fixed-point refinement. If $x^{\left(k\right)}$ denotes the current table at iteration k,m denotes the observation mask, and $x^{\text{obs}}$ denotes the measured data table, then one refinement step is conceptually

$$x^{\left(k+1\right)}=m⊙x^{\text{obs}}+\left(1-m\right)⊙G\left(E\left(x^{\left(k\right)}\right)\right).$$

Here, ⊙ denotes elementwise multiplication, so that observed entries remain clamped to measured values while only missing entries are updated. In AirQualityUCI, this refinement helps produce multivariate gap-filling estimates that can differ substantially from simple local linear interpolation, as illustrated in Fig. S5. For the *Drosophila* dataset, iterations stopped when the maximum absolute change over previously missing coordinates fell below $\varepsilon_{\text{imp}}=10^{-3}$ or after three passes, whichever occurred first. This refinement stage was skipped for fully observed data.

For the *Drosophila* dataset, we considered three initialization schemes for missing gene entries before VAE-based refinement:

1. **Zero-fill:** set missing gene-expression entries to 0 at the current time.
2. **Mean-fill:** for each positional bin and gene, set missing entries in that bin to the mean of that gene’s observed values in that bin.
3. **Random-fill:** initialize each missing entry by a uniform draw within the observed range of that gene.

As summarized in Supplementary Fig. S7, we compared these three initialization schemes under multiple latent dimensions and under two training objectives: the full five-term loss and a reduced objective omitting the decoder-smoothness and directional-alignment components. Random-fill initialization consistently yielded the best held-out reconstruction performance, with lower mean absolute error (MAE), lower mean squared error (MSE), and higher coefficient of determination ($R^{2}$) than zero-fill or mean-fill. Zero-fill performed worst across latent dimensions. For this reason, random-fill initialization was used in subsequent *Drosophila* experiments unless otherwise noted.

### 4.5 Latent neural ordinary differential equation

#### 4.5.1 Continuous-time latent dynamics

After learning a latent representation, we model temporal evolution in latent space using a neural ODE. If $z(t)\in R^{d_{z}}$ denotes the latent state at time t, then the latent dynamics are

$$\frac{dz}{dt}=f_{\theta }\left(z\left(t\right),u\left(t\right),t\right),$$

where $f_{\theta }$ is a neural network parameterized by θ. If no control variables are used for a given experiment, the equation reduces to

$$\frac{dz}{dt}=f_{\theta }(z(t),t)\;\text{or simply}\;\frac{dz}{dt}=f_{\theta }(z(t))$$

depending on whether explicit time dependence is included in the implementation.

Given an initial latent state $z\left(t_{0}\right)$, numerical integration produces latent trajectories z(t) for future times. These latent trajectories are then mapped back to observation space.

#### 4.5.2 Decoded rollouts

The first observation-space realization is a decoded rollout. After integrating the latent ODE, we decode the resulting latent path:

$$\overset{ˆ}{x}\left(t\right)=G\left(z\left(t\right)\right).$$

This yields a predicted trajectory directly in observation space. Decoded rollouts are useful when one wants smooth reconstructed trajectories or multi-step forecasts.

#### 4.5.3 Decoder pushforward

The second observation-space realization is the decoder pushforward. By differentiating the decoder output with respect to time, we obtain

$$\frac{d}{dt}G\left(z\left(t\right)\right)=J_{G}\left(z\left(t\right)\right)\frac{dz}{dt},$$

where $J_{G}(z)\in R^{d_{x}\times d_{z}}$ is the Jacobian of the decoder with respect to the latent state. Substituting the latent vector field gives

$$\frac{dx}{dt}=J_{G}\left(z\left(t\right)\right)f_{\theta }\left(z\left(t\right),u\left(t\right),t\right).$$

This induced observation-space velocity field is useful when one wants local derivatives directly in the original variables, for example for pollutant-space intervention screens or mechanistic supervision in gene space.

#### 4.5.4 Training objectives for latent dynamics

The latent neural ODE was trained to make short-horizon or interval-wise latent predictions consistent with encoder-derived latent targets. The exact loss form depends on the dataset. Conceptually, if $z_{t}^{\text{enc}}$ denotes the encoded latent state at time t and ${\tilde{z}}_{t+\Delta t}$ is the latent state obtained by integrating the neural ODE from t to t+Δt, then a typical latent prediction loss is

$${ℒ}_{\text{dyn}}={\left\|{\tilde{z}}_{t+\Delta t}-z_{t+\Delta t}^{\text{enc}}\right\|}_{2}^{2}.$$

When the downstream objective is defined in observation space rather than latent space, an additional decoded or pushforward loss may be used. The exact weighting of these terms, together with regularization, learning-rate schedules, and training-stage structure, is given in Tables S1–S3.

### 4.6 Dataset-specific methods for EEG

#### 4.6.1 EEG representation learning

For motor-imagery electroencephalography (EEG), each input consists of a short local window of 64-channel EEG centered at a given sample. Unless otherwise noted, each time point was represented by a 9-sample × 64-channel window, corresponding to 56 ms of data at 160 Hz, and each window was flattened to a 576-dimensional input vector. Windows were constructed with stride 1 for the default reconstruction and short-horizon dynamical analyses, and channel-wise normalization was applied before model fitting.

The universal variational autoencoder (VAE) was trained on pooled windows from multiple subjects and all run types to learn a shared representation of short-term EEG structure. The VAE itself does not take run identity as an input. Its role is to provide a subject-agnostic latent representation that can later be reused for forecasting, subject adaptation, and run-conditioned counterfactual analyses.

In the main EEG instantiation, the universal VAE used fully connected encoder and decoder networks with two hidden layers of width 512 and latent dimension $d_{z}=8$. The encoder produced the mean and log-variance of a diagonal Gaussian latent posterior, and the decoder mapped latent vectors back to the 576 reconstructed window entries. Training used a β-VAE objective with $\beta =3\times 10^{-4}$.

In reconstruction analyses, the decoder reconstructs the full local window. To align reconstructions with the original EEG time axis, we use the decoded center sample of each reconstructed window. This yields a sample-aligned reconstructed signal for comparison with the raw data. Unless otherwise stated, the trained universal VAE was then frozen for the subsequent EEG latent neural ordinary differential equation (neural ODE) experiments.

The subject split used in the experiments reported here was 70 subjects for training, 10 for validation during VAE training, and 29 held out for downstream evaluation and subject-adaptation experiments.

#### 4.6.2 Subject adaptation

To evaluate transfer and reuse of the learned EEG representation, we performed subject-level fine-tuning on held-out subjects. A pretrained universal VAE was fine-tuned on varying numbers of subject-specific windows while a fixed subject-specific test set was kept unchanged across adaptation budgets. This design isolates the effect of adaptation data availability from changes in the evaluation set. Reconstruction improvement after fine-tuning was used as the main metric.

#### 4.6.3 EEG latent dynamics and controls

For EEG dynamics, the latent neural ODE receives a run-level control vector u encoding the experimental run identity. The control represents contextual information about the task condition and is not reconstructed by the VAE. Instead, it enters only at the dynamical stage, allowing the same encoded EEG state to be rolled forward under different run conditions.

In the main EEG experiments, each run was encoded as a 7-dimensional one-hot control vector $u\in R^{7}$, corresponding to the seven protocol-defined run types R01–R07. The latent dynamics were modeled as

$$\frac{dz}{dt}=f_{\theta }\left(z\left(t\right),u\right),$$

with $z(t)\in R^{8}$. Here $f_{\theta }(z,u)$ was implemented as a multilayer perceptron taking the concatenated latent state and control as input, with three hidden layers of width 128, tanh activations, and an 8-dimensional output matching the latent dimension.

Short-horizon prediction is performed by encoding a window center into latent space, integrating forward for a prescribed number of steps, and returning the result to EEG space. In the pushforward formulation, the observation-space velocity is

$$\frac{dx}{dt}=J_{G}\left(z\left(t\right)\right)f_{\theta }\left(z\left(t\right),u\right),$$

where G denotes the decoder and $J_{G}(z)$ its Jacobian with respect to the latent state z.

For the EEG latent ODE experiments reported in the main text, training used short segments of length h=9 steps, corresponding to 9 sample intervals at 160 Hz. The loss penalized the discrepancy between the predicted latent state and the encoder-derived latent target at the end of each segment, with a modest additional emphasis on the final horizon step. During training, latent trajectories were integrated using an adaptive Dormand–Prince solver.

#### 4.6.4 Open-loop forecasting on EEG

For EEG forecasting benchmarks, we evaluated multi-step open-loop rollouts, meaning that after initialization the model predicts recursively without resetting to future ground-truth observations. Prediction accuracy was measured in EEG space by mean-squared error averaged over channels and held-out sequences. We compared the latent-dynamics backbone against a first-order vector autoregressive baseline and a persistence baseline. The exact forecast horizons, window lengths, strides, and train/validation/test splits are listed in Tables S1–S3.

#### 4.6.5 EEG counterfactual rollouts

Counterfactual EEG analyses were performed by holding the initial encoded state fixed and changing only the run-level control. If $u_{\text{base}}$ denotes the baseline control and $u_{\text{alt}}$ the alternative control, then two rollouts are generated from the same initial latent state $z_{0}$:

$$\frac{dz_{\text{base}}}{dt}=f_{\theta }\left(z_{\text{base}}(t),u_{\text{base}}\right),\;\frac{dz_{\text{alt}}}{dt}=f_{\theta }\left(z_{\text{alt}}(t),u_{\text{alt}}\right).$$

The final observation-space predictions are then compared through quantities such as

$$\Delta x_{\text{final}}=x_{\text{alt}}-x_{\text{base}}.$$

This isolates the effect of changing task context while keeping the initial EEG state fixed.

For the short-horizon EEG visualizations shown in the main text, we used a computationally efficient rollout protocol tailored to the displayed 9-step horizon. Given a window center, we first encoded the flattened window to obtain the latent initial condition $z_{0}\in R^{8}$. We then decoded the center sample to obtain the initial EEG state $x_{0}\in R^{64}$ and evaluated the decoder Jacobian $J_{G}\left(z_{0}\right)\in R^{64\times 8}$, where each entry is the partial derivative of one decoded center-channel value with respect to one latent coordinate.

Although the latent neural ODE was trained using an adaptive solver, the visualization itself used a fixed-step explicit Euler approximation because the displayed horizon was only 9/160 ≈ 56 ms. Specifically, with Δt=1 in sample units and fixed run-level control u, we advanced the latent state by

$$z_{k+1}=z_{k}+\Delta t\;f_{\theta }\left(z_{k},u\right).$$

We then mapped the latent slope into EEG space using the decoder Jacobian evaluated at the initial latent state,

$${\dot{x}}_{k}=J_{G}\left(z_{0}\right)\;f_{\theta }\left(z_{k},u\right),$$

and advanced the EEG-space trajectory by

$$x_{k+1}=x_{k}+\Delta t{\dot{x}}_{k}.$$

Because the displayed horizon was extremely short, this fixed-step protocol closely tracked the short-time behavior of the adaptive-solver model while allowing repeated rollouts over many windows at low computational cost.

### 4.7 Dataset-specific methods for AirQualityUCI

#### 4.7.1 Air-quality representation learning under missingness

For AirQualityUCI, each input is a short multivariate window centered at a target hour. The VAE receives pollutant and associated contextual measurements after lightweight preprocessing and masking. Because missingness is substantial, the reconstruction loss is computed only on measured entries. This encourages the model to learn from actual observations while still producing full reconstructed vectors.

The center of each reconstructed window is used as the denoised local state for downstream analysis. Post-VAE reconstructions are also used as refined imputations for entries missing in the raw table.

#### 4.7.2 Air-quality controls

The latent neural ODE for AirQualityUCI is conditioned on time-dependent external variables. These include calendar encodings and meteorological covariates. If u(t) denotes the concatenated control vector at time t, then the latent dynamics are

$$\frac{dz}{dt}=f_{\theta }\left(z\left(t\right),u\left(t\right),t\right).$$

The exact components of u(t), such as hour encodings, day encodings, weekend indicators, temperature, and other meteorological measurements, are listed in Tables S1–S3.

#### 4.7.3 Piecewise re-anchored observation-space trajectories

To generate day-scale pollutant trajectories while avoiding cumulative drift, we used piecewise reanchoring across local segments. Within each segment, the latent ODE is integrated, then mapped to observation space through either decoded rollouts or pushforward velocities. At the start of the next segment, the trajectory is re-initialized from the local observed or reconstructed state. Conceptually, if the segment boundaries are $t_{0}<t_{1}<\cdots <t_{K}$, then for each interval [$t_{k},t_{k+1}$] we initialize from a local state $x\left(t_{k}\right)$, encode it to $z\left(t_{k}\right)$, integrate the latent ODE over the interval, and map back to observation space.

This procedure keeps long trajectories stable while still using the continuous-time latent vector field within each segment. In pollutant space, the pushforward velocity is

$$\frac{dx}{dt}=J_{G}\left(z\left(t\right)\right)\;f_{\theta }\left(z\left(t\right),u\left(t\right),t\right).$$

#### 4.7.4 Air-quality forecasting baselines

For AirQualityUCI forecasting benchmarks, we compared the latent-dynamics backbone against persistence, a linear vector autoregressive model, a control-augmented vector autoregressive model, and a seasonal autoregressive integrated moving average model with exogenous regressors. Evaluation was performed using masked mean-squared error on measured pollutant entries only. Forecasts were generated in open-loop fashion for fixed horizons. All hyperparameters and fitting details are summarized in Tables S1–S3.

#### 4.7.5 Air-quality intervention screens

To probe the learned dependence on exogenous inputs, we performed *in silico* interventions by altering selected components of the control vector while keeping the initial state fixed. For example, if u(t) contains a temperature entry $u_{T}(t)$, then a temperature-swap intervention replaces $u_{T}(t)$ by an alternative value while keeping the other control components unchanged. The resulting change in instantaneous pollutant tendencies is

$$\Delta \dot{x}(t)=J_{G}(z(t))\;f_{\theta }\left(z(t),u_{\text{alt}}(t),t\right)-J_{G}(z(t))\;f_{\theta }\left(z(t),u_{\text{base}}(t),t\right).$$

This provides a local intervention screen over a scientifically interpretable control dimension.

### 4.8 Dataset-specific methods for the *Drosophila* atlas

#### 4.8.1 Atlas representation learning

For the *Drosophila* blastoderm atlas, each sample consisted of a current-time gene-expression vector together with optional spatial coordinates. When spatial context was included, we denote the gene-expression vector by $g_{t}\in R^{G_{\text{gene}}}$ and the spatial coordinates by $s\in R^{3}$, so that the encoder input was the concatenated vector

$$\left[g_{t}\|s\right]\in R^{G_{\text{gene}}+3},$$

where ‖ denotes concatenation. The atlas is temporally sparse and contains structured block-missing measurements across genes and times. The variational autoencoder (VAE) was trained with mask-aware reconstruction so that observed gene values contribute to the loss while missing entries do not. When spatial coordinates were included, they served as static contextual inputs attached to each cell or positional bin and were not reconstruction targets.

The encoder E produced a diagonal-Gaussian latent posterior with mean $\mu \in R^{d_{z}}$ and standard deviation $\sigma \in R^{d_{z}}$, equivalently diagonal variance $\sigma^{2}$. A latent sample was formed by the standard reparameterization

$$z=\mu +\sigma ⊙\epsilon ,\;\epsilon \sim 𝒩\left(0,I\right),$$

where ⊙ denotes elementwise multiplication. The decoder G then mapped the latent state back to a gene-space prediction ${\overset{ˆ}{g}}_{t}\in R^{G_{\text{gene}}}$.

The reconstruction term averaged masked squared error over a mini-batch, and the Kullback–Leibler (KL) term used the standard VAE divergence together with a warm-up weight. Using the notation of this manuscript, the KL term was

$${ℒ}_{\text{KL}}=\frac{1}{2}\sum_{j=1}^{d_{z}}\left(\mu_{j}^{2}+\sigma_{j}^{2}-\text{log}\;\sigma_{j}^{2}-1\right),$$

and its training weight followed a linear warm-up schedule

$$w_{\text{KL}}=\beta \cdot \text{min}\left(1,\frac{\text{epoch}+1}{\text{warmup}}\right),$$

where β is the target KL weight and warmup is the number of epochs over which the KL term is ramped from zero to its full value. As throughout the manuscript, the same per-sample gene mask was applied to all gene-level loss terms so that unobserved or placeholder-filled entries never contributed gradients.

For the *Drosophila* VAE, we additionally used mild regularization terms to encourage decoder smoothness and a simpler latent-to-gene map when the learned observation-space vector field would later be used to supervise a mechanistic student model. These regularizers were introduced because the downstream teacher–student transfer depends not only on reconstruction quality, but also on the local geometric behavior of the decoder G and the stability of the induced observation-space dynamics. The additional terms included a decoder-Jacobian penalty, which discourages excessively irregular local variation in the decoder map; an optional local directional-alignment term, which encourages consistency between nearby latent displacements and their decoded gene-space directions; and a soft sparsity penalty on network weights, which favors a simpler latent-to-gene mapping.

Thus, in the regularized *Drosophila* setting, the full VAE objective took the form

$$ℒ={ℒ}_{\text{recon}}+w_{\text{KL}}{ℒ}_{\text{KL}}+\lambda_{J}{ℒ}_{\text{Jac}}+\lambda_{\text{dir}}{ℒ}_{\text{dir}}+\lambda_{\text{sp}}{ℒ}_{\text{sp}},$$

where ${ℒ}_{\text{recon}}$ is the masked reconstruction loss, ${ℒ}_{\text{KL}}$ is the KL divergence, ${ℒ}_{\text{Jac}}$ is the decoder-smoothness penalty, ${ℒ}_{\text{dir}}$ is the optional directional-alignment term, and ${ℒ}_{\text{sp}}$ is the soft sparsity penalty. These additional terms were used only in the relevant *Drosophila* experiments and were kept mild so as not to dominate the reconstruction objective.

Decoder smoothness was controlled by a Jacobian-size proxy that estimated the squared norm of the decoder Jacobian applied to random probe directions. Specifically,

$${ℒ}_{\text{Jac}}=E_{v}{\left\|J_{G}(z)v\right\|}_{2}^{2}\approx \frac{1}{P}\sum_{p=1}^{P}{\left\|\frac{G\left(z+\varepsilon v_{p}\right)-G\left(z-\varepsilon v_{p}\right)}{2\varepsilon }\right\|}_{2}^{2},$$

where $v_{p}$ are Hutchinson probe vectors, P is the number of probes, and ε>0 is a small finite-difference step. When a true next developmental time point was available, the optional directional term encouraged the decoder pushforward to align with the observed short-time gene change:

$${ℒ}_{\text{dir}}=1-\text{cos}\;\left(J_{G}\left(z\right)r,\Delta g\right),\;r=\frac{\mu_{t+1}-\mu_{t}}{\left\|\mu_{t+1}-\mu_{t}\right\|},\;\Delta g=g_{t+1}-g_{t}.$$

This term was evaluated only when a valid next time point existed and, in practice, only when ‖Δg‖ exceeded a small threshold so that nearly zero changes did not dominate the alignment score. The soft sparsity term ${ℒ}_{\text{sp}}$ applied gentle shrinkage to network weights. Small positive coefficients $\lambda_{J},\lambda_{\text{dir}}$, and $\lambda_{\text{sp}}$ weighted the Jacobian, directional, and sparsity terms, respectively. Their exact formulas and hyperparameter settings are reported in Supplementary Tables S1–S2.

To visualize the structure of the learned latent space, we fit a Uniform Manifold Approximation and Projection (UMAP) embedding on the latent encodings of all positional bins and then fixed this embedding across time. The resulting embedding, shown in Supplementary Fig. S6, separates developmental stages while preserving continuity across the latent manifold, illustrating that the VAE captured both spatial and temporal structure in a form suitable for downstream dynamical modeling.

#### 4.8.2 Latent dynamics for temporally sparse gene-expression data

After VAE training, each developmental time point was mapped into latent space. For the *Drosophila* atlas, the latent neural ordinary differential equation (neural ODE) was then trained on consecutive latent pairs obtained by encoding the refined data at successive developmental times. In practice, we used the encoder mean $\mu_{t}$ as the deterministic latent state associated with time t. Because the dataset has only six time points, the neural ODE is used mainly to provide smooth latent interpolation, denoised local derivative estimates, and short-horizon predictions rather than dense long-horizon forecasting of the kind used in EEG.

The latent vector field $f_{\theta }$ was parameterized by a fully connected multilayer perceptron with three hidden layers and output dimension $d_{z}$. Numerical integration was performed with torchdiffeq using the adaptive Dormand–Prince method (dopri5). The latent neural ODE was differentiated through this adaptive solver using automatic differentiation. For *Drosophila*, we used stricter solver tolerances than in the EEG experiments because the developmental dynamics were more sensitive to numerical integration error. Exact solver implementations and tolerance settings are provided in the Supplementary Information.

For *Drosophila*, the latent neural ODE was trained with an uncertainty-weighted endpoint loss that down-weighted latent coordinates whose encoder posterior variance was larger. Using the notation of this manuscript, if ${\overset{ˆ}{z}}_{k+1}$ denotes the latent state predicted by integrating the neural ODE to the next developmental time point and $z_{k+1}$ denotes the corresponding encoder-derived latent target, then the loss was

$${ℒ}_{\text{dyn}}^{\text{var}}=E\left[\sum_{j=1}^{d_{z}}\frac{{\left({\overset{ˆ}{z}}_{k+1,j}-z_{k+1,j}\right)}^{2}}{\sigma_{k+1,j}^{2}+\varepsilon }\right],$$

where $\sigma_{k+1,j}$ is the encoder-predicted posterior standard deviation for latent coordinate j at developmental time $t_{k+1}$, and ε>0 is a small constant for numerical stability. This weighting reduces the influence of endpoint coordinates that are more uncertain under the encoder posterior and was used because developmental interpolation in the atlas was more sensitive to latent uncertainty than the denser EEG setting.

Training and validation examples for the latent neural ODE were constructed from consecutive developmental-time pairs and then split into training and validation subsets for model selection.

Two observation-space realizations were used as teacher signals for downstream mechanistic modeling.

##### Pushforward realization.

Starting from a state encoded from a measured or reconstructed gene-expression vector, we compute the observation-space velocity

$$\frac{dx}{dt}=J_{G}\left(z\left(t\right)\right)f_{\theta }\left(z\left(t\right),t\right).$$

This starts exactly from the local state used for initialization and avoids reconstruction mismatch at the segment boundary.

##### Decoded-rollout realization.

We also integrate the latent path z(t) over an interval, decode it to obtain a dense gene-expression trajectory

$$\overset{ˆ}{x}\left(t\right)=G\left(z\left(t\right)\right),$$

and then estimate derivatives from that decoded path on a fine grid. This provides smooth within-interval teacher trajectories. The two realization modes are therefore related but not identical.

#### 4.8.3 Teacher signals in gene space

The black-box backbone supplies two types of signals needed for mechanistic fitting.

First, it supplies refined gene-expression tables through mask-aware reconstruction and optional fixed-point refinement. For the refined *Drosophila* data used in teacher construction, the encoder was applied deterministically using the latent mean only, with stochastic sampling and dropout disabled. Second, it supplies local gene-space dynamics, either through decoder pushforward or through derivatives estimated from decoded latent rollouts. These teacher signals are used to supervise a mechanistic student model described below.

### 4.9 Mechanistic student model in gene space

#### 4.9.1 Why we adopted a Hill-type student model

After obtaining black-box observation-space dynamics for the *Drosophila* atlas, we initially explored symbolic regression as a possible route to an explicit interpretable model. In principle, symbolic regression could discover closed-form governing equations directly from the teacher-provided derivatives. In practice, however, unconstrained symbolic regression frequently produced expressions containing unstable division terms and mismatched polynomial degrees in numerators and denominators. Although such expressions could fit local derivatives, they often became numerically unstable when integrated over the developmental time scales of interest.

We therefore adopted a predefined mechanistic hypothesis class, namely a Hill-type gene regulatory model over $G_{\text{gene}}$ genes, and fit its parameters to the black-box teacher signals. This choice traded some functional flexibility for improved numerical stability and direct biological interpretability. The overall teacher–student framework is not restricted to Hill functions, but in this study the Hill model provided a stable editable interface for equation-level perturbation experiments.

#### 4.9.2 Hill-type regulatory ordinary differential equation

For mechanistic distillation in the *Drosophila* analyses, we used a Hill-type ordinary differential equation in gene space. Let $g(t)\in R_{\geq 0}^{G_{\text{gene}}}$ denote the gene-expression vector, and let $g_{i}(t)$ denote the expression of target gene i, with $i=1,\ldots ,G_{\text{gene}}$. The mechanistic student takes the form

$$\frac{dg_{i}}{dt}=\alpha \left(b_{0,i}+\sum_{j=1}^{G_{\text{gene}}}V_{ij}H\left(g_{j};K_{ij},n_{ij}\right)-\gamma_{i}g_{i}\right),\;H\left(g;K,n\right)=\frac{g^{n}}{K^{n}+g^{n}},$$

where:

- α>0 is a global production scale,
- $b_{0,i}$ is the basal term for gene i,
- $V_{ij}$ is the directed interaction weight from regulator j to target i,
- $K_{ij}>0$ is the Hill midpoint,
- $n_{ij}>0$ is the Hill exponent,
- $\gamma_{i}>0$ is the linear decay coefficient for gene i.

To keep notation consistent with the Results, we denote the signed interaction matrix by V, where $V_{ij}$ is the fitted signed influence of regulator j on target i. Positive $V_{ij}$ indicates activation and negative $V_{ij}$ indicates repression within the chosen mechanistic hypothesis class. We write

$$\theta_{\text{Hill}}=\left\{\alpha ,b_{0},V,K,n,\gamma \right\}$$

for the full set of Hill-model parameters.

Thus, the student model contains two layers of parameters:

1. a sparse signed interaction structure V,
2. continuous kinetic and shape parameters such as $\alpha ,\gamma_{i},b_{0,i},K_{ij}$, and $n_{ij}$.

#### 4.9.3 Teacher-guided and data-only fitting regimes

We considered two mechanistic fitting regimes.

##### Teacher-guided regime.

The student is fit using black-box-derived gene-space supervision signals. The learned model, referred to as the black-box teacher, consists of the VAE encoder/decoder paired with a latent neural ODE. Consistent with the main-text neural ODE section, we form teacher gene-space derivatives in two ways: (i) a *pushforward gene-space* ODE, where the latent vector field is mapped through the decoder Jacobian to obtain a gene-space velocity at the observed state and we integrate piecewise starting from the measured $g\left(t_{k}\right)$; and (ii) *decoded latent rollouts*, where we integrate the latent ODE across each interval, decode a dense path in gene space between $t_{k}$ and $t_{k+1}$, and compute derivatives along that path by finite differences on a fine grid. In both constructions, any spatial coordinates s are held fixed and are not integrated. When either of these teacher-derived velocities is used during training, we refer to the regime as *teacher-guided*.

##### Data-only regime.

The student is fit without black-box teacher guidance, using targets derived directly from observed data under the corresponding comparison protocol. In this case, derivatives come only from finite differences on the original developmental sampling grid. We refer to this regime as *data-only*.

These two regimes allow us to test whether the black-box teacher genuinely improves mechanistic fitting or merely provides a more elaborate preprocessing stage. Both regimes use the same Hill-model class, sparsity penalties, pruning threshold, and calibration strategy.

##### Interpretation of interventions.

Because the atlas contains only wild-type embryos and no matched perturbation measurements, inferred Hill couplings should be interpreted as candidate directed interactions consistent with the learned dynamics, rather than as formally established causal effects. Interventions in this study therefore correspond to structural edits of the fitted equations, used to generate *in silico* mechanistic hypotheses.

#### 4.9.4 Training objective, sparsity pruning, and calibration

Training pairs ($g_{t},g_{t+1}$) were obtained from consecutive developmental measurements. The Hill-type mechanistic student was fit under either teacher-guided or data-only supervision. In the data-only regime, target derivatives were approximated by finite differences on the original developmental sampling grid. In the teacher-guided regime, target derivatives were instead provided by the black-box teacher velocities, using either decoder-pushforward velocities or finite differences along decoded latent rollouts.

The fitting objective combined three terms. First, a derivative-matching loss aligned the Hill right-hand side with the target derivative, using either teacher-informed velocities in the teacher-guided regime or finite-difference estimates in the data-only regime. Second, a one-step prediction loss was computed by numerically integrating the Hill ODE from t to t+1 and comparing the predicted state with the observed next state $g_{t+1}$. Third, a collocation-style consistency term encouraged agreement between the integrated trajectory and the differential equation at intermediate points. An $\ell_{1}$ penalty on the interaction matrix V was included throughout training to promote sparsity in the inferred regulatory graph.

The relative weighting between derivative supervision and one-step prediction was scheduled over training. Early epochs emphasized derivative matching, whereas later epochs emphasized one-step prediction, with cosine blending between two user-defined milestones ($E_{0},E_{1}$). For sparsity regularization, we explored $\lambda_{\ell_{1}}\in \left\{10^{-3},10^{-4}\right\}$. Optimization used Adam with gradient clipping. The learning rate followed a warm-up phase up to epoch ep_maxlr, followed by logistic decay. Numerical integration of the Hill system during fitting used 5 substeps per unit developmental interval.

To promote interpretability, we imposed an $\ell_{1}$ penalty on V and then hard-pruned weak couplings after the initial fit. Specifically, after the initial training stage, entries satisfying

$$\left|V_{ij}\right|<10^{-3}$$

were removed. The surviving interaction support

$$ℰ=\left\{(i,j):V_{ij}\neq 0\right\}$$

defines the retained directed gene–gene graph. After pruning, the interaction support ℰ was frozen, and the sign pattern of the retained $V_{ij}$ entries was also preserved.

A calibration stage was then performed to refine the continuous parameters while holding the learned graph structure fixed. In this stage, we retained the learned interaction support ℰ and the corresponding sign pattern of $V_{ij}$, and adjusted only the remaining dynamical parameters on the retained graph. Specifically, calibration refined the global production scale α, the per-gene decay rates $\gamma_{i}$, the basal terms $b_{0,i}$, and the Hill constants and exponents ($K_{ij},n_{ij}$) for nonzero edges (i,j)∈ℰ.

In one implementation, these remaining parameters were refined sequentially: first α, then $\gamma_{i}$, followed by optional production rescaling, then $b_{0,i}$, and finally $\left(K_{ij},n_{ij}\right)$ on (i,j)∈ℰ. Each calibration stage optimized one-step forecast mean-squared error while applying a mild quadratic penalty ($\lambda =10^{-3}$) only to the parameters being tuned at that stage, so that previously tuned parameters remained close to their current values. Numerical integration during calibration also used 5 substeps per unit developmental interval. Thus, calibration preserved the sparse learned regulatory topology while improving quantitative one-step predictive accuracy through refinement of the retained kinetic and regulatory parameters.

#### 4.9.5 Mechanistic predictions and interventions

Once fitted, the Hill student can be numerically integrated forward in time from a chosen initial state. This enables both forecasting and intervention analysis.

##### Gene knockout.

A gene knockout is implemented by forcing a selected gene level to zero during integration or by modifying the corresponding state-update rule according to the experiment.

##### Single-edge edit.

A specific regulatory edge can be removed by setting

$$V_{ij}=0$$

for a chosen regulator–target pair while leaving all other parameters unchanged.

##### Graded edge scaling.

A coupling can also be weakened or strengthened by scaling it:

$$V_{ij}\leftarrow \lambda V_{ij},$$

where λ is a prescribed scaling factor. This yields dose-response-style *in silico* perturbation predictions.

Because these edits operate directly on explicit equations, the mechanistic student provides an interpretable interface for dynamical hypothesis generation. At the same time, we do not interpret these edits as proving formal causal identification from observational data alone. Rather, they define testable mechanistic hypotheses within the fitted model class.

### 4.10 Teacher–student comparison protocols

#### 4.10.1 Comparison on fully observed genes

To compare teacher-guided and data-only mechanistic fitting under matched conditions, we restricted certain analyses to subsets of genes with complete measurements across all time points. This avoids confounding from missing ground truth and ensures that one-step prediction targets are comparable between regimes. For these experiments, we evaluated one-step forecasts from t to t+1 on fixed train/test partitions using identical sparsity and calibration procedures in both regimes.

#### 4.10.2 Full 99-gene setting

We also returned to the full gene space, where missingness is substantial. In this regime, VAE-based reconstruction and optional fixed-point refinement provide the missing values needed to construct derivative estimates and one-step targets across all genes. Thus, even when teacher guidance does not always yield the lowest direct mechanistic prediction error, the black-box stage remains essential because it makes full-network mechanistic fitting feasible under partial observation.

### 4.11 Training procedures and optimization

#### 4.11.1 Stagewise training

The framework was trained in stages. First, the VAE was trained to convergence or near convergence using mask-aware reconstruction and KL regularization. Second, latent states derived from the trained VAE were used to train the latent neural ODE. Third, in the *Drosophila* analyses, the mechanistic student was fit using the resulting teacher signals.

This stagewise design improves optimization stability and makes the role of each model component easier to interpret. Where alternative joint or partially coupled training variants were explored, those details are summarized in Tables S1–S3 and in the Supplementary Information.

#### 4.11.2 Optimization and numerical integration

All neural components were optimized with gradient-based stochastic optimization. The precise optimizer, learning rate, batch size, epoch count, and early-stopping or checkpointing choices vary by dataset and experiment and are reported in Tables S1–S3.

Latent and mechanistic ODEs were integrated numerically using standard ODE solvers. Solver tolerances, maximum-step settings, and related numerical choices are reported in Tables S1–S3 where relevant. For all experiments, the same solver family was used consistently within a comparison so that differences reflect model structure rather than numerical-method changes.

#### 4.11.3 Validation and model selection

Model selection was performed using validation losses appropriate to each stage. For VAEs, validation was based on reconstruction-oriented criteria. For latent neural ODEs, validation was based on latent prediction error, decoded forecast error, or the experiment-specific dynamical loss used in training. For the mechanistic student, selection considered prediction quality together with sparsity and interpretability constraints. When multiple checkpoints or random seeds were explored, the chosen model was selected from the corresponding validation criterion described in the experiment tables.

### 4.12 Model selection and evaluation overview

All neural-network components in this work, including encoders, decoders, and latent neural ordinary differential equations, were trained by stochastic gradient-based optimization with automatic differentiation. For each stage, model selection was based on held-out validation performance using the experiment-appropriate validation loss. Unless otherwise noted, we report results from the checkpoint with the best validation performance. Exact optimizer choices, learning-rate schedules, batch sizes, and other stage-specific hyperparameters are summarized in Tables S1–S3.

For EEG, open-loop forecasting performance was evaluated in EEG space as mean-squared error (MSE) averaged across held-out sequences and all 64 channels. Universal-representation adaptation was evaluated on the 29 held-out subjects using fixed subject-specific test sets and varying fine-tuning budgets. Short-horizon control perturbations held the initial latent state fixed and altered only the run-identity control.

For AirQualityUCI, forecasting performance was reported as masked MSE computed only on pollutant entries measured at the target time. We used both a random block split and a seasonal 24-hour-block evaluation protocol. For intervention experiments, the initial state was held fixed while selected control components were changed, for example by replacing January temperature inputs with July temperatures at matched day and hour.

For the *Drosophila* atlas, unless otherwise noted, experiments used the fixed 90/10 positional-bin split with 608 held-out bins. Teacher-guidance comparisons were restricted to the 27 genes measured at all six time points to avoid confounding by imputed ground truth. Mechanistic performance was assessed by one-step forecasts and by trajectory-level comparisons under baseline and edited equations.

### 4.13 Evaluation metrics

#### Mean-squared error.

For forecasting and reconstruction in EEG and AirQualityUCI, the primary metric was mean-squared error (MSE). If $\overset{ˆ}{x}$ is a prediction and x the target, then

$$\text{MSE}=\frac{1}{d_{x}}\sum_{i=1}^{d_{x}}{\left({\overset{ˆ}{x}}_{i}-x_{i}\right)}^{2}.$$

When masks are present, the metric is computed only on observed entries.

#### Masked mean-squared error.

If $m_{i}\in \{0,1\}$ indicates whether entry i is observed, then the masked MSE is

$${\text{MSE}}_{\text{masked}}=\frac{\sum_{i}m_{i}{\left({\overset{ˆ}{x}}_{i}-x_{i}\right)}^{2}}{\sum_{i}m_{i}+\varepsilon }.$$

#### Relative error for gene-expression prediction.

For some *Drosophila* analyses, we used the relative error

$$\frac{\left|g-\overset{ˆ}{g}\right|}{|g+\overset{ˆ}{g}|+\varepsilon },$$

where g is the ground-truth expression and $\overset{ˆ}{g}$ is the predicted value. This reduces domination by highly expressed genes and improves comparability across genes with different dynamic ranges.

### 4.14 Interpretational scope

The latent-dynamics backbone is a black-box predictive model that supports forecasting, controlled rollouts, and observation-space intervention screening. The mechanistic student is an interpretable dynamical approximation derived from the teacher signals. In the *Drosophila* analyses, the resulting sparse regulatory graph is therefore interpreted as a set of candidate directed influences consistent with the learned dynamics in the observed regime. Equation-level edits of the Hill student define mechanistic perturbation hypotheses that can be compared against published experiments or tested in future perturbation studies. They should not be interpreted as proving formal causality from observational data alone.

### 4.15 Implementation summary

All dataset-specific architecture choices, latent dimensions, control encodings, training schedules, regularization strengths, pruning thresholds, calibration settings, and solver parameters are listed in Tables S1–S3. The main text emphasizes the shared conceptual pipeline, while these tables and the Supplementary Information provide exact reproducibility details for each dataset and training stage.

## Supplementary Material

Document S1, including Figures S1–S14, Tables S1–S3, and supplemental references.

Supplementary Files

This is a list of supplementary files associated with this preprint. Click to download.

- Suppfinalreduced.pdf
- FoundationModelNPJSBAsupp.zip

## Figures and Tables

**Table 1.** One-step forecast errors and nonzeros after pruning ($\left|V_{ij}\right|<10^{-3}$). “Non-zero edges” counts $|ℰ|=\#\left\{(i,j):V_{ij}\neq 0\right\}$. Teacher-guided models are as good or better; the advantage widens under stronger sparsity.

Setting	Regime	MAE with SD	MSE	Non-zero edges
$\lambda_{\ell_{1}}=10^{-3}$	Teacher-guided	**0.05716**±0.02134	**0.00716**	151
$\lambda_{\ell_{1}}=10^{-3}$	Data-only	0.05958±0.02209	0.00775	15
$\lambda_{\ell_{1}}=10^{-4}$	Teacher-guided	**0.05239**±0.01820	**0.00592**	≈232
$\lambda_{\ell_{1}}=10^{-4}$	Data-only	0.05279±0.01893	0.00600	≈289

## Data Availability

All experiments were implemented in Python, with neural-network training based on PyTorch Lightning and ODE integration based on torchdiffeq. Figure generation and figure assembly were performed in Python and MATLAB. Reproducible code for preprocessing, model training, and evaluation is available at https://github.com/nihcompmed/drosobb2mech. All datasets analyzed in this study are publicly available from the cited sources. No new human or animal data were generated in this work.
